# A Deformable Generic 3D Model of Haptoral Anchor of Monogenean

**DOI:** 10.1371/journal.pone.0077650

**Published:** 2013-10-28

**Authors:** Bee Guan Teo, Sarinder Kaur Dhillon, Lee Hong Susan Lim

**Affiliations:** Institute of Biological Science, Faculty of Science, University of Malaya, Kuala Lumpur, Malaysia; University of Nottingham, United Kingdom

## Abstract

In this paper, a digital 3D model which allows for visualisation in three dimensions and interactive manipulation is explored as a tool to help us understand the structural morphology and elucidate the functions of morphological structures of fragile microorganisms which defy live studies. We developed a deformable generic 3D model of haptoral anchor of dactylogyridean monogeneans that can subsequently be deformed into different desired anchor shapes by using direct manipulation deformation technique. We used point primitives to construct the rectangular building blocks to develop our deformable 3D model. Point primitives are manually marked on a 2D illustration of an anchor on a Cartesian graph paper and a set of Cartesian coordinates for each point primitive is manually extracted from the graph paper. A Python script is then written in Blender to construct 3D rectangular building blocks based on the Cartesian coordinates. The rectangular building blocks are stacked on top or by the side of each other following their respective Cartesian coordinates of point primitive. More point primitives are added at the sites in the 3D model where more structural variations are likely to occur, in order to generate complex anchor structures. We used Catmull-Clark subdivision surface modifier to smoothen the surface and edge of the generic 3D model to obtain a smoother and more natural 3D shape and antialiasing option to reduce the jagged edges of the 3D model. This deformable generic 3D model can be deformed into different desired 3D anchor shapes through direct manipulation deformation technique by aligning the vertices (pilot points) of the newly developed deformable generic 3D model onto the 2D illustrations of the desired shapes and moving the vertices until the desire 3D shapes are formed. In this generic 3D model all the vertices present are deployed for displacement during deformation.

## Introduction

One of the problems encountered in studying structural and functional morphology of small organisms, in particular parasites such as the monogeneans (Platyhelminthes), is the lack of visualisation in three dimensions. Images for 3D visualisation can be obtained using hardware such as confocal microscope [1], [2], Scanning Electron Microscope (SEM) [3], Transmission Electron microscope (TEM) [4], Position Electron Microscopy (PET), micro-ct scan [5] and software such as Amira [6], Bioptonics 3001 OPT [7] and Imaris (for serial histological sections) [8], but these hardware and software are usually expensive. Many of these instruments also require complicated and tedious preparations prior to usage, for example, confocal microscopy requires special stains [1] and micro-ct scan [5] requires the specimens to be held immobilised and that media used to hold these specimens should not be denser than the specimens, which for transparent flatworms will be a challenge.

The 3D images generated using hardware can provide information on the structural aspects but most of these images cannot be manipulated to simulate functions since these 3D images are basically static. Leadbeater et al. [3], however, were able to combine 3D images from SEM and Java programming to obtain graphical (digital) models of the choanoflagellates. A digital 3D model, on the other hand, will allow both visualisation in three dimensions as well as interactive manipulation [6], [9], [10] (Table S1). 3D models have also been developed for the creation of physical model of a human skull and a human brain through 3D printing [11] (Table S1). Developing digital 3D models for micro-organisms could be easier, less time consuming and do not require any special preparation skills or expensive software or hardware, but it is necessary and pertinent to possess a good knowledge of the morphological structures of the organisms in question, good quality 2D illustrations and an in-depth knowledge of the 3D development tools. There would be the initial investment of time to study and understand the technical skills and principals of geometry involved in designing 3D models. The rationale for developing a 3D model from 2D illustration is because most of the structural information on species are 2D illustrations obtained from light microscopy study and these 2D illustrations serve as a rich source of 2D templates for developing 3D structures [12].

### The organism of interest: Monogenea Carus, 1863

Prior to exploring methods to be used in constructing a generic 3D model which can be deformed into different shapes, we would like to briefly describe the subject of this study, the monogeneans, and the rationale for developing deformable 3D models for the various hard parts of the monogeneans. Briefly the monogeneans (Class Monogenea, Phylum Platyhelminthes) can be divided into 3 subclasses viz. Polystomatoinea Lebedev, 1986, Oligonchoinea Bychowsky, 1937 and Polyonchoinea Bychowsky, 1937 [13]. They are hermaphrodites possessing both male and female reproductive organs, simple alimentary, ladder-like nervous system and haptoral attachment organs. They are softbodied flat worms which cannot withstand desiccation and have lengths ranging from about less than 1 mm to about 3 mm for most dactylogyrideans [14], [15] to 6 mm for frog polystomes [16] and from 1.2 cm to about 3.2 cm for *Oculotrema* sp. (Polystomatidae) of hippopotamus [17]. The male and female reproductive systems are made up of both soft anatomical structures as well as sclerotised hard parts (male copulatory organs and female vaginal system). The haptors of the monogeneans consist of a great diversity of sclerotised and unsclerotised armaments which are involved in the attachment of monogeneans onto the various organs of their hosts such as gills of fishes, urinary bladder of frogs and conjunctiva of turtles and hippopotamus [14], [15], [16], [17], [18]. The sclerotised haptoral armaments range from one or two pairs of anchors, 14 or 16 marginal hooks and usually one or two connective bars or bars absent and squamodiscs. Non-sclerotised parts include clamps and suckers [16], [17], [18] and even the haptor itself can function as clasping organ [15], [19], [20]. Basically the polyonchioneans possess anchors, bars, marginal hooks, with or without squamodiscs and with or without suckers as in *Diplectanocotyla* (see [21]). Whilst the polystomatineans as represented by the polystomes possess anchors, marginal hooks and suckers [16], [17], [18]. The oligochoineans (such as diplozoids and microbothriids) possess suckers, clamps, anchors, marginal hooks and sclerites associated with suckers [22], [23].

In this paper we will be focusing on the anchors of the monogeneans belonging to the Order Dactylogyridea (Polyonchoinea). The dactylogyrideans usually have 14–16 marginal hooks, 2 to 4 anchors and 0, 1, 2 to 3 bars as well as sclerotised and unsclerotised male and female reproductive organs. Characteristics such as shapes and sizes and the relative positions of the various organs of an organism are important diagnostic characters in taxonomic description and identification. In the monogeneans, the sclerotised hard parts of the haptor and of the male and female reproductive parts are of diagnostic importance and are used for species identification (see [14]–[24]). The main concern in using these hard parts as taxonomic characters is that the shapes of these hard diagnostic parts can be distorted by preparation methods and by the way the specimens are orientated on the glass slides [25]. This is why Lim & Gibson [25] strongly recommended that specimens are properly depressed with the right amount of pressure to best expose and orientate the structures on lateral sides for a standardised measurement. In this study we used 2D illustrations of anchors which are disposed on their lateral side.

We are interested in the functions of the sclerotised hard parts of this diverse group of platyhelminth. The elucidation of the morphology and functions of these structures in the monogeneans is not easy due to the fragile nature of their neodermis which cannot withstand desiccation and long investigations under the microscope. We therefore decided to explore the use of digital 3D models to help us to understand the morphology as well as the functions of the different morphological structures. The versatility of 3D models makes them suitable and useful tools in education and research especially to elaborate how different organs in an organism function and to elucidate the overall morphology from different viewing angles [12]. By manipulating 3D models of these diagnostic features, for instance, one can ascertain whether the observed shapes and sizes are true shapes or are due to preparatory methods (see [12]). 3D models of anchor, marginal hook and bar had been developed in our previous study but they were not deformable [12] (see Discussion). Our aim here is to develop a generic 3D model which can be reused to form 3D shapes of different species without going through the tedious task of developing 3D models from scratch.

### Terms used

It is necessary here to define what we mean by points and point primitives and other related terms used herein and by others in geometric constructions of 3D models. A point is the simplest most primitive of geometric object and it is the basic building block for all other geometric objects [26]. Point primitive is considered a basic geometric element by Fu & Shan [27]. The point and point primitive seems to denote the same thing. In this paper we will be using the terms point primitives as used by Fu & Shan [27]. Vertex (plural vertices) is a kind of point that describes the corners or intersections of geometric shapes [28]. In computer graphics a vertex is a point in both 2D and 3D space with coordinates [29]. 3D Cartesian coordinates are triplet numbers (x, y, z) specifying a location of a point in 3D space [30]. Deformation occurs when modification is made to a baseline geometry. The direct manipulation method is the displacement for a certain number of selected vertices on the surface of the geometry [31]. Different terms are used for the vertices selected for direct manipulation method, they are known as pilot points in Anderson et al. [31], as control points in Pourazady & Xu [32], and control vertices in Murakawa et al. [33]. Another commonly used term in morphometry is the landmark points or landmarks, which are “points in a shape object in which correspondences between and within the populations of the object are preserved and landmarks include vertices, anchor points, control points, sites, profile points, ‘sampling’ points, nodes, markers and fiducial markers” [34]. Therefore we would like to adopt the definition used by Li et al. [35] who considered landmark points as points assigned on the surfaces of 2D images or/and 3D model. However, it is not stated by these authors whether these landmark points are selected or not selected from vertices existing in 3D models for the deformation process. Control points were originally used by Sederberg & Parry [36] and later by Anderson et al. [31] for the external points (floating above the surface and not on the surface) that control the deformation of the shape in free form deformation technique. In this paper we would be using the term point primitives for the points defined on the surface of the 2D illustration during constructive modeling when the geometry is built from scratch and all the point primitives are denoted by Cartesian coordinates, which will become vertices in the constructed 3D model. In our 3D model all these vertices will become pilot points during direct manipulation deformation process. Some authors caused deformation by assigning landmarks (reflective points or contour points or fiducial points) on the 2D image which correspond to a set of vertices on the 3D model; the vertices on the 3D model are displaced according to the positions of the landmark points on the 2D template to form the new 3D form as depicted by the 2D template [37], [38], [39].

### Literature review

A literature review on the development of digital 3D models is done and the information is summarised in Table S1. This review does not include 3D imaging and images since we are interested in developing generic digital 3D model. Literature review shows that 3D models have been developed for biological specimens and innate objects mainly for 3D visualisation (static 3D models) using software such as Autodesk 3ds Max and for a lesser extent for manipulation and 3D printing (Table S1). The first 3D models of haptoral elements (anchors, marginal hooks and connective bars) of monogeneans were constructed using Autodesk 3ds Max for visualisation in three dimensions and for manipulation [12] but these 3D models are not deformable 3D models.

It is possible to create new 3D models based on existing 3D models in two main ways (Table S1). The first is by assigning landmark point (reflective marker or contour point) on the 2D template (target) and aligning the vertices of the existing 3D model to match the landmark points on the 2D template resulting in a 3D model for the target shape [37], [38]. Although Murakawa et al. [33] also assigned sketch points on 2D images but they selected vertices on existing 3D models as control vertices instead of using all the vertices. The second way to make existing 3D models deformable is by assigning landmarks points (fiducial nodes, feature points) on the existing 3D models & 2D images [35], and matching the landmark points on the both 3D models and 2D images. The landmark points on the 3D models can be moved to fill 2D targets to form new 3D shape. The technique of displacement of selected existing vertices on the surface of generic 3D model to give rise to different shapes is termed direct manipulation technique [31]. Based on this definition the deformation methods used by Tang & Hui [37], Kraevoy et al. [38] and Murakawa et al. [33] can be classified as direct manipulation technique. This technique allows a modeller to deform the shape of a generic 3D model in a more intuitive and user-friendly way because this technique focuses only on the control of a comparatively sparser collection of vertices on the surface of generic model [31].

### Objective

We have attempted to assign landmark points on our existing 3D anchor developed in Autodesk 3ds Max [12] but the resulting new 3D shapes were not satisfactory (see Discussion). Our main aim here is to develop a morphologically simple deformable generic 3D model of an anchor of the parasitic monogeneans which can be easily and rapidly deformed into different 3D anchor shapes, obviating the need to repeat tedious modelling process.

To develop the new deformable 3D anchor model, we adopt the geometric modelling technique based on point primitives used in architectural building design [27] to construct a stack of rectangular building blocks for the generic 3D model. The geometric modelling technique of using polyhedron building blocks [27] is adopted because of the fewer numbers of basic geometric elements (point primitives) in the polyhedron blocks necessary to construct a 3D model, in fact a rectangular block only has 8 point primitives, whilst rectangular surface has 4 point primitives. Since all these point primitives will be used as pilot points for deformation of the generic 3D models into different required shapes by altering the positions of these point primitives, the number of point primitives should not be too large to complicate deformation [40] nor too few so that complicated shapes cannot be formed [41] (see Discussion).

## Methodology

Briefly, the generic 3D model of an anchor will be developed by manually assigning a set of point primitives along the surface of the 2D anchor template during constructive modeling process. These point primitives are then grouped in the form of six polyhedron surfaces that constitute a rectangular building block. As already noted 3D rectangle is used as building block for our generic 3D model because only eight point primitives are needed to make a single rectangular building block. Instead of using complicated mathematic formula in analytic geometry to define the Cartesian coordinates (x, y, z) for each point primitive, the simpler graphical method is used here to assign the coordinates manually on a 2D anchor printed on a Cartesian graph paper [42], [43]. The rectangular building blocks can be stacked on top of each other following the position marked by the Cartesian coordinates of the point primitives until a 3D wireframe similar to the shape of the 2D template is obtained. To visualize the structure of the rectangular building blocks, 3D wireframes of the preliminary generic 3D model of anchor are developed using the Cartesian coordinates (given in Table S2) as parameters in the GraphicComplex function in Mathematica (an integrated technical computing software, version 8, http://www.wolfram.com/mathematica/) and also in Blender (an open source modelling tool version 2.63, http://www.blender.org/blenderorg/blender-foundation/) for comparative purposes.

To construct and generate the preliminary generic 3D model of the anchor, the Cartesian coordinates of the point primitives for each rectangular block stack in the 2D template are written into the Python scripts in Blender to form the 3D geometry of the shape of the 2D anchor template. Since the present 3D geometry consists of rectangular building blocks which are not able to represent curvature and cylindrical shapes, the 3D model developed has angular edges. One way to overcome the angularities is to use Catmull-Clark subdivision surface modifier, a smoothening tool in Blender to smoothen the 3D geometry to obtain a smoother and more natural shape [44]. The number of point primitives in the preliminary generic 3D model is next optimized to produce the final generic 3D model to ensure that proper deformation to different diverse shape. To deform the resulting final generic 3D model into different anchor shapes, all the vertices on the 3D model are used as pilot point and deformation is effected by moving the original positions of the pilot point (essentially changing the Cartesian coordinates) to fit the new 2D shape.

### A. Selecting a 2D template and determining sites of high morphological variations by analysing different monogenean anchors

**Figure 1:**
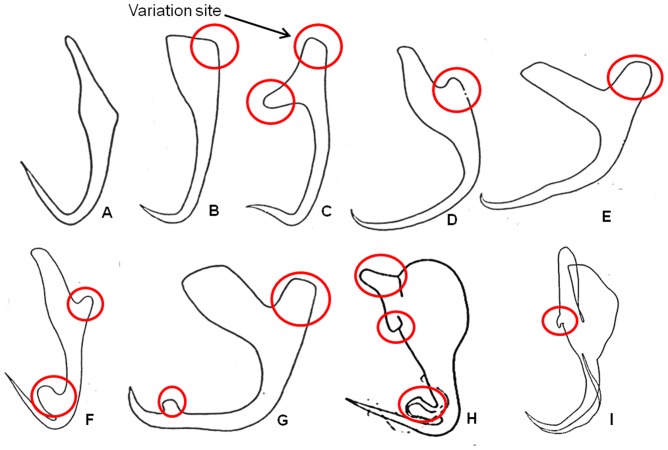
2D illustrations of selected monogenean anchors from publications [20], [24]. (A) *Bivaginogyrus obscurus* Gusev, 1955 (selected as 2D template for generic 3D model). (B) *Dactylogyrus primarius* Gusev, 1955. (C) *Pellucidhaptor merus* Zaika, 1961. (D) *Dactylogyrus falcatus* Wedl, 1857. (E) *Dactylogyrus vastator* Nybelin, 1924. (F) *Dactylogyrus pterocleidus* Gusev, 1955. (G) *Dactylogyrus falciunguis* Achmerow,1952. (H) *Chauhanellus auriculatum* Lim, 1994. (I) *Chauhanellus caelatus* Lim, 1994 (red circles denote sites of high morphological variations).

Prior to starting the process of developing a deformable generic 3D model we need to select a 2D illustration of an anchor of a dactylogyridean monogenean which is simple and uncomplicated to be used as a 2D template. It is done by reviewing the different publications on monogeneans species belonging to the dactylogyrideans to extract all possible monogenean anchors types (Fig. 1) [see 20, 24]. It is also necessary that the 3D model be deformed into all possible morphological diversities (see Fig. 1) using direct manipulation deformation method. Therefore these anchors are also examined to determine the locations or sites of high morphological variations such as “bulges”, ornamentations and “extrusion” (Figs. 1B–1I). We noted seven sites on the anchors where most morphological variations are likely to occur (Figs. 1B –1I). This information will be used in optimisation process (see Section E below). After analysing the different anchors we selected the morphologically simple anchor of *Bivaginogyrus obscurus* Gusev, 1955 as our 2D template (Fig. 1A) (see [20]).

### B. Assigning point primitives on 2D template on Cartesian graph paper and extracting Cartesian coordinates for point primitives using graphical method for preliminary generic 3D model

**Figure 2:**
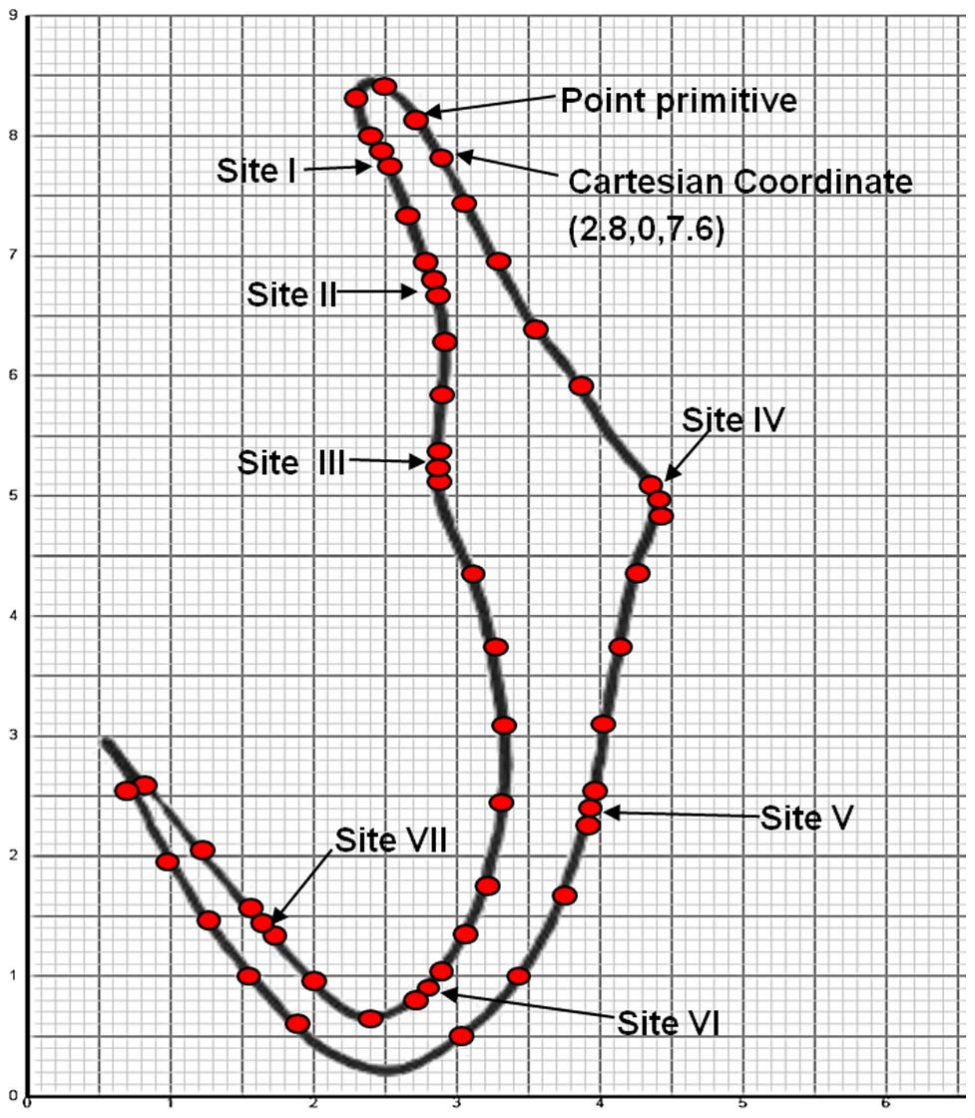
2D illustration of anchor of *Bivaginogyrus obscurus* printed on a Cartesian graph. Dots represent positions of point primitives manually assigned along the anchor outline (preliminary model). (More point primitives are allocated in the sites of high morphological variations (Sites I–VII) in the final 3D model: see Fig. 6).

The selected 2D anchor template of *B. obscurus* (Fig. 1A) is printed on a Cartesian graph paper. A set of point primitives (to form rectangular building blocks) are manually assigned along the anchor outline. More point primitives (instead of 4 point primitives at normal sites) are added and stacked at the 7 sites noted to have high morphological variations in the anchor (Fig. 2) to allow for more deformation of the final generic 3D model. The number of additional point primitives to be assigned to the sites of high morphological variations is determined through the process of trial and error by increasing the number of point primitives on the preliminary generic model by an addition of 2 point primitives until we obtained the desired shape of the most diverse anchor (see Section E below).

The Cartesian graph paper allows each of the point primitive to be manually defined by a set of Cartesian coordinates (x, y, z) (Fig. 2) which are important parameters to construct rectangular building block for 3D model. These Cartesian coordinates are stored in Microsoft Word file (Table S2). The Cartesian coordinates will be used to build the wireframes of the preliminary generic model of 3D anchor in Mathematica and in Blender and to build the 3D geometric model (rectangular building blocks) in Blender.

### C. Constructing 3D wireframes for preliminary generic 3D model using Mathematica and Blender

**Figure 3:**
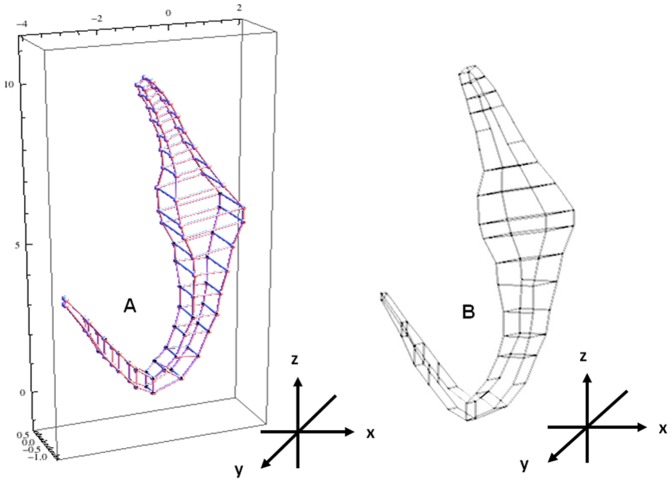
3D wireframe of preliminary generic 3D anchor. (A) 3D wireframe constructed in Mathematica. (B) 3D wireframe constructed in Blender.

The construction of the wireframe is to visualise the initial underlying structure of the 3D model by displaying its rectangular building blocks (skeleton). The wireframes of the preliminary generic 3D model are constructed using both Mathematica and Blender (Fig. 3A– 3B) to determine which platform will provide a better wireframe model. The Cartesian coordinates obtained from the graph (Table S2) are used as parameters in the GraphicComplex function in Mathematica to develop the wireframe for the preliminary generic 3D model (Fig. 3A) (see Text S1 for codes). A Python script is written in Blender using the Cartesian coordinates from the graph paper (see Text S2 for script; Table S2 for coordinates) to produce another wireframe for the preliminary generic 3D model (Fig. 3B) for comparative purpose.

### D. Constructing 3D rectangular building blocks and developing preliminary generic 3D model in Blender

**Figure 4:**
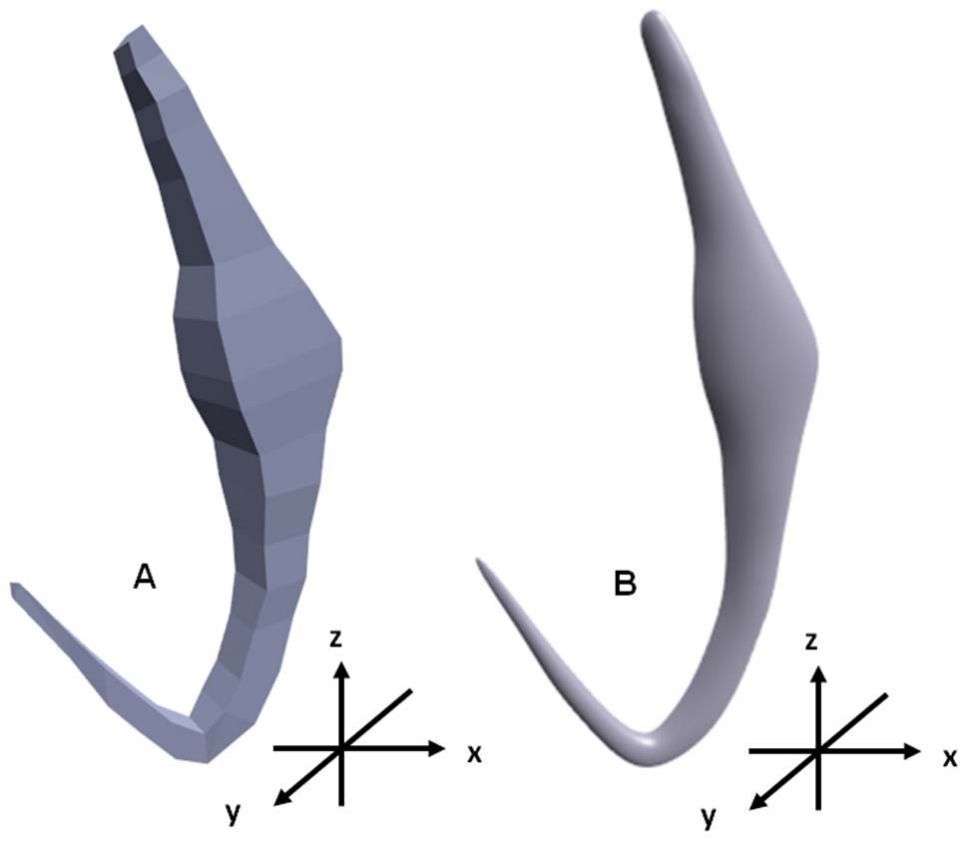
Preliminary generic 3D model of anchor. (A) Before smoothening. (B) After smoothening using Catmull-Clark subdivision surface modifier and anti-aliasing rendering option in Blender.

A Python script (see Text S3) is written using the Python Application Programming Interface in Blender to construct 3D rectangle as the building blocks for the preliminary generic 3D model. The Cartesian coordinates for each rectangular block are used as parameters in the Python scripts to form the 3D geometry of the anchors (Fig. 4A).

The Catmull-Clark subdivision surface modifier (see Text S4), a smoothening tool in Blender, is used to smoothen the angular 3D geometry of the resulting 3D model. Jagged edges along the newly developed 3D model are reduced by rendering it using anti-aliasing option in Blender to obtain a smoother preliminary generic 3D model of anchor with less jagged edge (Fig. 4B). However these jagged edges cannot be completely eliminated (see Discussion).

### E. Optimization of number of point primitives on the preliminary generic 3D model to cater for deformation at sites of high morphological variations

The basic number of point primitives for a rectangular surface is 4. To ensure that proper deformation of the generic 3D model to different diverse shape can occur there is a need to increase the number of point primitives at certain sites on the model especially at sites of high morphological variations (Fig. 2). However there need to be a balance in the number of point primitives used because too many point primitives will make deformation tedious and complicated since too many points will have to be used in the deformation process. Hence before arriving at the final generic 3D model we need to add in the optimal number of point primitives at sites of high morphological variations.

**Figure 5:**
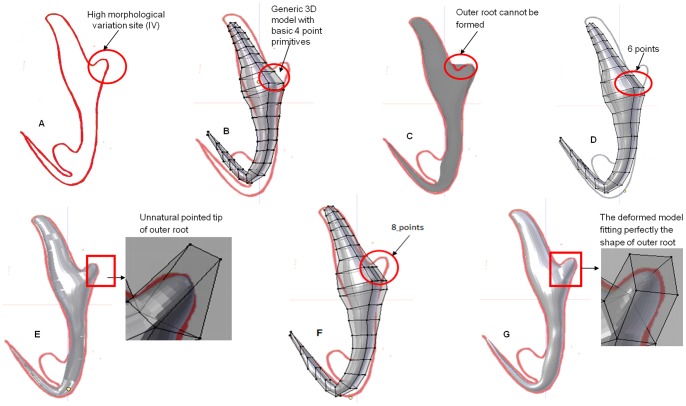
Process of optimization of the number of point primitives in sites of high morphological variations. (A) 2D illustration of anchor of *Dactylogyrus pterocleidus* (target shape, outlined in red). (B) Preliminary generic 3D model (showing point primitives in rectangular blocks) with 4 point primitives at Site IV, overlaid on 2D target shape. (C) Derived 3D model (incomplete, in grey) of target shape based on 4 point primitives. (D) Preliminary generic 3D model assigned with 6 point primitives at Site IV and 2D target shape. (E) Derived 3D model (incomplete, in grey) of target shape based on 6 point primitives. (F) Preliminary generic 3D model assigned with 8 point primitives at Site IV and 2D target shape. (G) Derived 3D model (incomplete, in grey) of target shape based on 8 point primitives.

The positions and amount of point primitives are important to ensure effective and accurate construction of the generic 3D model for deformation into different desired shapes [45]. We have to ensure that the numbers of point primitives used are not too high or too few to provide better control in the deformation process and to avoid complication during deformation and at the same time ensuring that there are enough point primitives in the 3D model so that it can be deformed into all the possible anchor shapes. To optimize the number of point primitives, we first examined all the known diverse anchors (Fig. 1) to determine the degree of variation a particular site is likely to have (see above). The preliminary generic 3D model (Fig. 4B) developed in Blender without any additional point primitives will be used in the optimisation of point primitives. In this optimisation process the target anchor used is *Dactylogyrus pterocleidus* Gusev, 1955. The target anchor is imported into Blender (Fig. 5A) and the preliminary generic 3D model is aligned onto it for deformation by direct manipulation to derive the 3D anchor (Fig. 5B) (see Section G(ii)). The number of point primitives is increased at Site IV by 2 until we are able to deform the outer root (Site IV) to obtain the desired target shape and size (Figs. 5D & 5F). The optimum numbers of point primitives which are necessary to deform each of the sites to attain the most diverse shape are given in Table 1.

**Table 1:** Number of point primitive on each site of high morphological variation as indicated in Fig. 1.

Sites of morphological variations	Number of point primitives
Sites without variations	4
I	8
II	8
III	8
IV	8
V	6
VI	16
VII	16

The preliminary 3D model with 4 point primitives at site IV could not be deformed to obtain the desired shape of the outer root of the 3D anchor model for *D. pterocleidus* (Fig. 5C). There are not enough point primitives to deform to fit the target shape (Fig. 5C). To develop the outer root we needed 8 point primitives at Site IV to obtain the outer root although the outer root is morphologically a simple structure (Fig. 5G). The same process is done for all the other 6 sites (Fig. 2). As expected the sites having higher morphological variations have higher number of point primitives (Table 1). For example at Site VI, 16 point primitives are needed to deform and create the ‘ear’ *of D. pterocleidus* as well as *C. auricalatum* (Fig. 1H) (see later).

### F. Formation of final deformable generic 3D model

**Figure 6:**
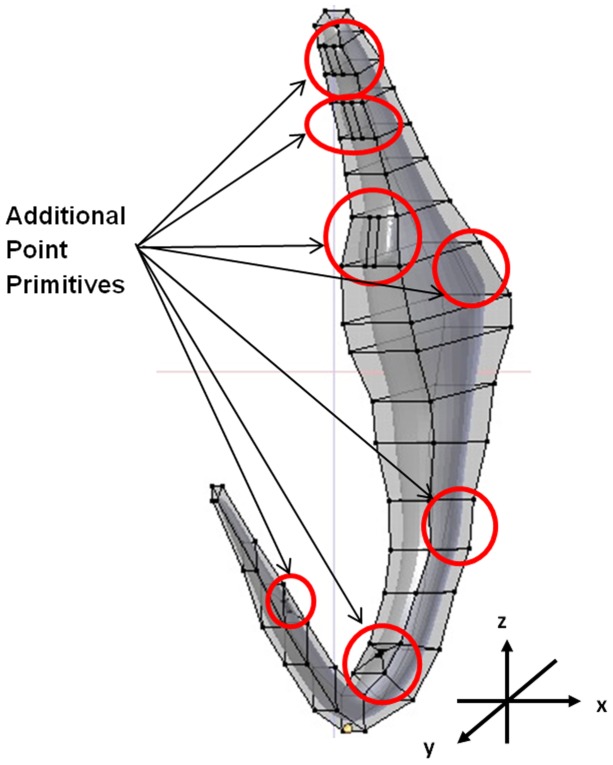
Final deformable generic 3D model of anchor after inclusion of point primitives in all sites of high morphological variations and after smoothening using Catmull-Clark subdivision surface modifier and anti-aliasing option in Blender.

The optimal numbers of point primitives obtained from the above optimisation process are added onto the graphical 2D template and the Cartesian coordinates obtained for these point primitives. These new coordinates (Table S3) are fed into previous Python script of the preliminary 3D model (Text S4) and a new set of script is obtained (Text S5). By executing the final set of script the final deformable generic model is generated (Fig. 6).

### G. Deformation into different desired 3D shapes

In this study we have deformed the generic 3D models into desired 3D shapes in two ways: (1) by changing the Cartesian coordinates of the preliminary generic 3D model, and (2) by direct manipulation of the final generic 3D model. This is done to compare the quality of the 3D model derived from these two methods.

#### (i) Deformation through changing Cartesian coordinates of the preliminary 3D model in Python script

Deformation of the 3D model to derive new 3D shapes is done by changing the Cartesian coordinates in the preliminary generic 3D model in Blender. This method of deriving the new shape requires a repeat of the whole process of printing out the new shape on graph paper, finding and optimising the necessary point primitives to be added to the graphical 2D template, manually calculating the Cartesian coordinates and putting these coordinates into the Python script of the preliminary generic 3D model (Text S4).

We elaborate this process by selecting the anchor of *Dactylogyrus vastator* Nybelin, 1924 (Fig. 1E). The selected 2D illustration of the target or desired shape is first printed on a graph paper. The Cartesian coordinates are manually obtained for the 7 sites of high morphological variations as noted in Fig. 2. These coordinates (Table S4) are entered into the source code of the preliminary generic 3D model (Text S4) to change the coordinates in the generic 3D model in Blender (See Text S6).

#### (ii) Deformation of final deformable generic 3D model into different desired shapes through direct manipulation deformation method in Blender

**Figure 7:**
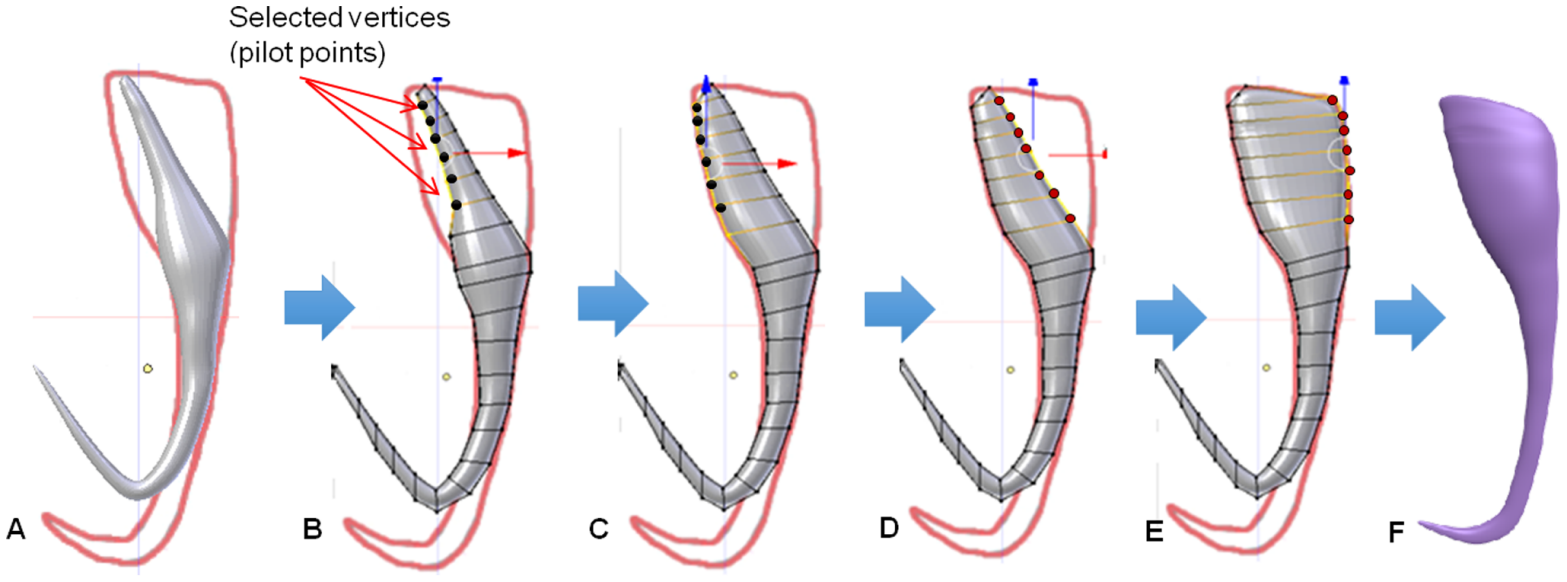
Process of deformation of final generic 3D model into desired shape by direct manipulation deformation technique. (A) 2D illustration of *Dactylogyrus primarius* (in red) with final generic 3D model (in grey). (B) Pilot points on final generic 3D models are selected. (C) Selected pilot points are moved to fill the 2D shape. (D–E) Other selected pilot points are moved to fill up the whole 2D shape. (F) New derived 3D shape.

**Figure 8:**
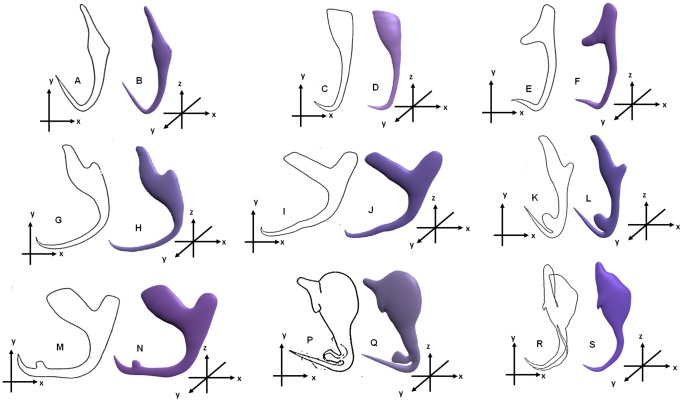
2D anchor templates (unshaded) and corresponding 3D models (coloured). (A–B): 2D anchor template & final deformable generic 3D anchor model of *Bivaginogyrus obscurus*. (C–D): *Dactylogyrus primarius*. (E–F): *Pellucidhaptor merus*. (G–H): *Dactylogyrus falcatus*. (I–J) *Dactylogyrus vastator*. (K–L): *Dactylogyrus pterocleidus*. (M–N): *Dactylogyrus falciunguis*. (P–Q) *Chauhanellus auriculatum*. (R–S) *Chauhanellus caelatus*.

The newly developed final deformable generic 3D model with the extra point primitives added to each site of high morphological variation (Fig. 6) is used to generate 3D models for other anchor shapes. The deformation process used here is the direct manipulation method and it is employed here to develop 3D models of the 8 different anchor shapes (Fig. 1B–1I). To do this the 2D illustrations of the required new anchor (target) shapes are imported into Blender and the final deformable generic 3D model is aligned onto the 2D illustration (Fig. 7A) and deformed using direct manipulation deformation method by first selecting one or more vertices (pilot points) on the surface of final deformable generic 3D model (Fig. 7B) and moving them to fit the outline of the 2D illustration (Fig. 7C). The shape of deformable generic 3D model is deformed following the movement of selected vertices. The deformation is repeated by selecting other vertices and moving them to fill up the 2D illustration (Fig. 7D & Fig. 7E) to obtain the desired 3D shape (Fig. 7F). All the vertices in the final deformable 3D model are used in the deformation. The same deformation approach is also used for the optimisation process. In this study 3D anchors are derived from the final 3D model for 8 dactylogyridean species, viz. *Dactylogyrus primarius* Gusev, 1955, *Pellucidhaptor merus* Zaika, 1961, *Dactylogyrus falcatus* Wedl, 1857, *Dactylogyrus vastator* Nybelin, 1924, *Dactylogyrus pterocleidus* Gusev, 1955, *Dactylogyrus falciunguis* Achmerow, 1952, *Chauhanellus auriculatum* Lim, 1994 and *Chauhanellus caelatus* Lim, 1994 (Fig. 8).

**Figure 9:**
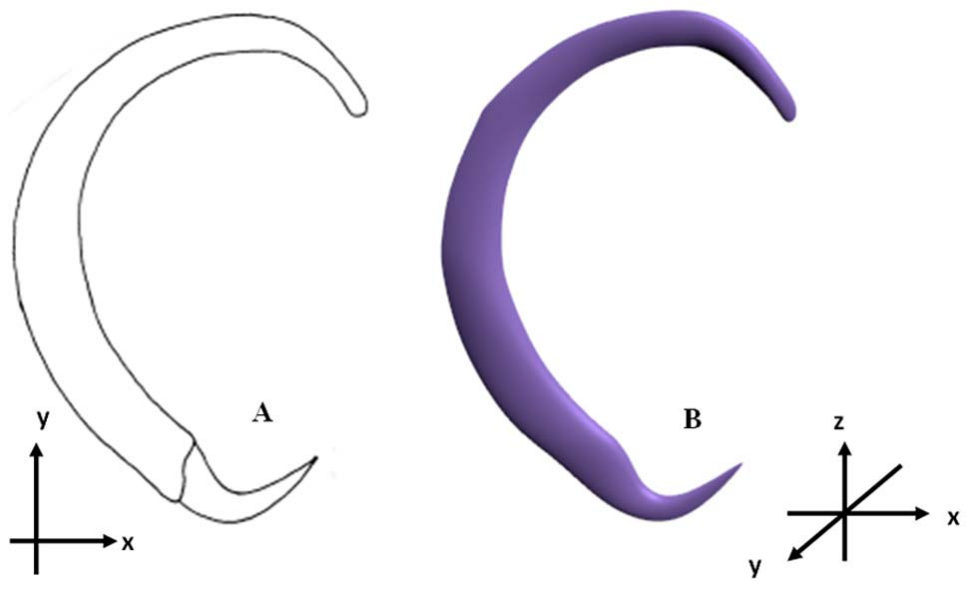
2D illustration and 3D model of hook-like sclerite of *Squalonchocotyle mitsukurii* Kitamura, Ogawa, Taniuchi & Hirose, 2006 derived from the final deformable generic 3D anchor model by direct manipulation deformation method. (A) 2D illustration of sclerite. (B) 3D model of sclerite.

To show the versatility of the final deformable generic 3D anchor model developed based on *B. obscurus*, a dactylogyridean, we have also deformed the final generic model to generate the hook-shaped sclerite of the terminal suckers of the hexabothriid, *Squalonchocotyle mitsukurii* Kitamura, Ogawa, Taniuchi & Hirose, 2006 (Fig. 9) [46].

**Figure 10:**
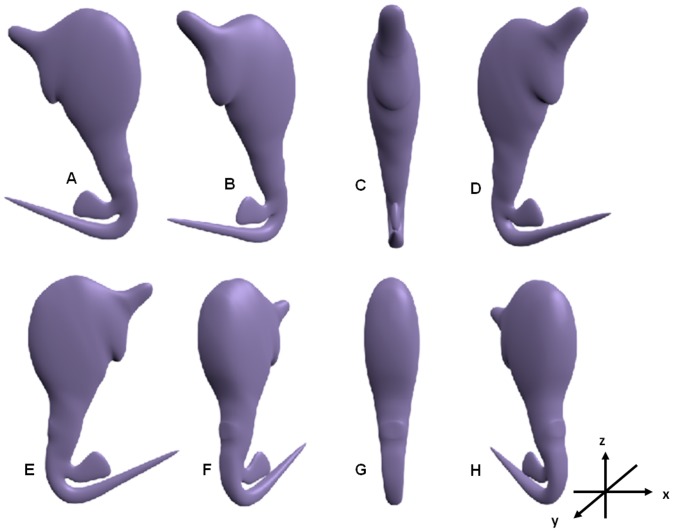
Views of the 3D anchor of *Chauhanellus auricalatum* in different degrees of rotation (anti-clockwise) in the x-axis. (A) Side view at 0° rotation. (B) At 45°. (C) At 90°. (D) At 135°. (E) At 180°. (F) At 225°. (G) At 270°. (H) At 315°.

The Cartesian 3D coordinates for each of the 3D shapes derived from the final deformable generic 3D model could be obtained from Transform Properties Window in Blender and given in Tables S5, S6, S7, S8, S9, S10, S11, S12 and S13. The newly derived 3D models of the different anchors can be rotated in Blender through 360° to reveal the views of the shape of the anchor in different orientations as exemplified in Figs. 10 for the 3D anchor of *C. auriculatum*.

**Figure 11:**
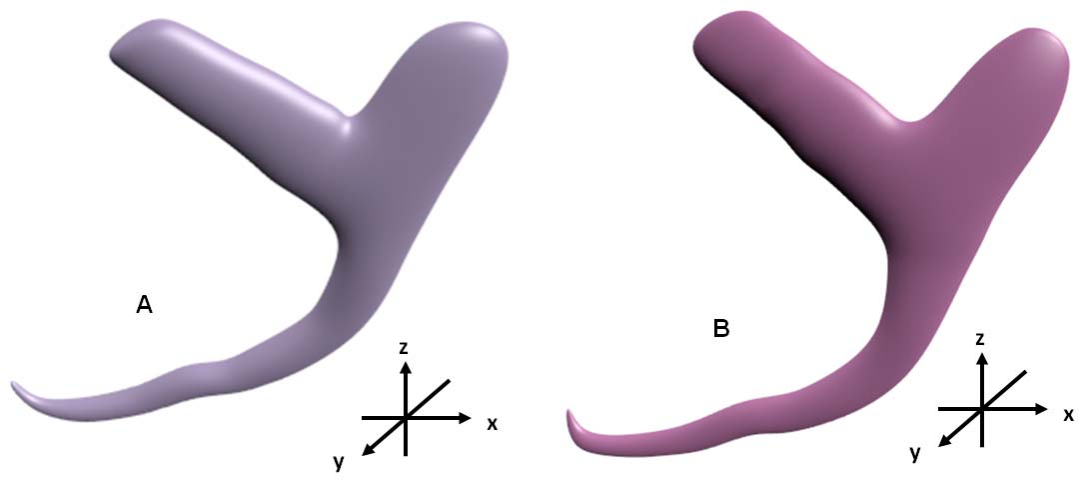
3D models of anchor of *Dactylogyrus vastator* derived from two deformation methods. (A) 3D model derived from changing Cartesian coordinates. (B) 3D model derived from direct manipulation method.

The derived 3D models resulting from changing the coordinates and the 3D anchor of *D. vastator* derived from direct manipulation method are compared (cf. Fig. 11A & Fig. 11B).

## Results

### 3D Wireframe models of preliminary generic 3D anchor

The two wireframe models produced indicate the outlines of the rectangular blocks used in constructing the preliminary generic 3D anchor model (Figs. 3A & 3B). The wireframe produced in Mathematica is better compared to that produced in Blender (cf. Fig. 3A & Fig. 3B). The lines in the wireframe of the 3D model produced in Mathematica are clearer and more defined than the wireframe model from Blender.

### Comparison of 3D anchors derived by changing Cartesian coordinates and by direct manipulation method

New 3D shapes can be derived by altering the coordinates of the preliminary generic 3D model (see Text S6) or through direct manipulation method of the final deformable generic 3D model. The 3D anchors derived for *D. vastator* by changing the coordinates (Fig. 11A) and by direct manipulation method (Fig. 11B) indicate no observable differences in the 3D shapes derived (cf. Fig. 11A & Fig. 11B). Of the two methods the direct manipulation method is simpler to use and does not require tedious calculation of Cartesian coordinates (see Methodology section). Besides having no noticeable advantage in using the latter method of deformation by changing coordinates, this process is also tedious and time consuming practically starting from scratch and any variations on subsequent new shapes have to be manually calculated, thus defeating the purpose in developing a rapid and accurate method for deriving new 3D shapes. The direct manipulation deformation of the final generic deformable 3D model to develop new derived 3D models is found to be rapid, more user friendly and less tedious and produces quality 3D shapes.

### 3D anchors derived by direct manipulation method

Figs. 8C–8S and Figs. 9A–9B show the nine original 2D illustrations of anchors of varying complexities and their derived 3D forms (Figs. 8D, 8F, 8H, 8J, 8L, 8N, 8Q, 8S & 9B) constructed by deforming the newly developed final deformable generic 3D anchor model (Fig. 8B) through direct manipulation method. The final deformable generic 3D anchor model could be deformed to form extrusions and morphologically different inner root as in Fig. 8L & Fig. 8N by simple direct manipulation method using all the vertices (pilot points). This simple deformable generic 3D anchor model (Fig. 6) can be deformed into complex anchor shapes because of the additional point primitives at the relevant sites on the 3D model. Seven sites have been identified (Fig. 2) by examining different anchors (Fig. 1). We have also obtained the 3D coordinates for the derived 3D anchors of the 9 monogenean species in this manuscript from Blender and have been included in the supplementary files as Tables S5, S6, S7, S8, S9, S10, S11, S12 and S13. The 3D Cartesian coordinates for the preliminary and final generic 3D anchor models are Tables S2 and S3. It should be noted that of the 10 monogenean species used in this study, 9 of them including the species for the generic model belong to the dactylogyridean monogeneans (Fig. 8) and only one, the hexabothriid species, belong to the oligochoinean monogenean (Fig. 9).

The final deformable generic 3D model and the derived new 3D shapes have been rotated through 360° to reveal their 3-dimensional nature. Herein only the rotated views for the 3D anchor model of *C. auriculatum* are shown (Fig. 10). Fig. 10 also shows the differences in shapes of the 3D anchor in different orientations. This further elaborates the versatility of the newly developed generic 3D model. It should be noted that the final deformable generic 3D model (Fig. 8B) and the derived 3D models (Figs. 8D, 8F, 8H, 8J, 8L, 8N, 8Q, 8S & 9B) are smoothened using the Catmull-Clark smoothen modifier in Blender to reduce the angularities caused by the rectangular building blocks. The jagged edges of the generic and derived 3D models caused by the inherent rendering problem in Blender are reduced using the antialiasing option in Blender but not completely eliminated.

## Discussion

In this paper, we have successfully developed a simple deformable generic 3D model for monogenean anchor, which can be deformed into different shapes (Fig. 8) using the direct manipulation deformation method (by moving the pilot points). We have shown that a deformable digital generic 3D model can be developed using relatively simple geometric construction method, with little complex mathematics and without using any high end computer technology and employing only simple point primitives, Cartesian graph paper and accessible software (Blender).

Our final deformable generic 3D model is visually simple (Fig. 6) but it can be deformed by direct manipulation deformation method (by displacing the vertices) into the different required anchor shapes and forms (Figs. 8C–8S) and all the vertices are deployed in the deformation. This generic 3D anchor model is not developed from existing 3D models and is also different from other deformable 3D models because it is developed from point primitives in Blender. In this 3D model all the vertices are used as pilot points during the deformation instead of selected vertices [33] making the model more deformable and versatile. Since all the vertices are to be used in deformation process we have been careful to ensure that not too many or too few point primitives are assigned: we have to balance the need for more point primitives at sites of high structural variations to allow for deformation into the diverse forms as shown in Fig. 8 and fewer vertices which will allow better control during deformation. Rectangular building blocks are used in constructing this deformable generic 3D model as indicated by the wireframes for the generic 3D model (Fig. 3A & Fig. 3B). The reason why we use rectangular building blocks despite their angularities is because basically such building blocks required the least number of point primitives.

### Comparison to other 3D models

As already noted some existing 3D models are made deformable by either selecting a certain number of pilot points from existing vertices [33] to match to the sketch points on the 2D template or assigning landmark points (fiducial nodes) on existing 3D models to match fiducial points assigned on 2D target image [35], [39]. This deformable generic 3D model is simpler and could be used without any matching of landmark points on target 2D illustrations unlike the deformable 3D car model developed by Kara & Shimada [39] which requires matching the selected fiducial nodes on the deformable 3D model to the fiducial points assigned on the 2D illustrations.

We have tried to make our previous 3D models built in Autodesk 3ds Max [12] deformable by assigning landmark points onto our previous 3D models as done by Kara & Shimada [39]. However we found that the resulting derived 3D shapes, are not satisfactory and not ‘natural’ and complex monogenean anchors such as the anchor of *C. auriculatum* cannot be formed. Besides, the deformations of the numerous vertices on the existing 3D model from Autodesk 3ds Max are difficult, clumsy and time consuming. This is why we opted to build a completely new 3D model. In comparison this newly developed deformable generic 3D model constructed by geometric rectangular building blocks give better derived 3D shapes than the 3D shapes derived using previous existing 3D models developed using cylindrical primitive shape in Autodesk 3ds Max.

### Future Improvements to generic 3D model

The use of stackable rectangular building blocks to construct the 3D model allows for greater freedom to choose the number and location of point primitives on the 2D shape or template. The degree of deformability in a generic 3D model can be altered by changing the number and location of point primitives. The increase in point primitives at the sites of high morphological variations, allows for greater deformation and for the generation of extrusions thus enabling the formation of special features on a monogenean anchor (Figs. 8K, 8M, 8P & 8R). We have shown in our results that the newly constructed deformable generic 3D model can be deformed into a variety of different 3D anchor shapes by directly moving the vertices on the surface of the generic 3D model (direct manipulation method) (see Fig. 7). The drawback in using rectangular building blocks is that the resulting 3D model looks angular despite using the smoothening tool, Catmull-Clark subdivision surface modifier in Blender, to smoothen the 3D model (see Fig. 4B). The rectangular building blocks alone are not to be able to completely approximate the more complex and complicated geometry of organisms. A possible solution to obtain a more natural shape is to combine primitive shape such as cylinder and sphere into a single 3D geometry instead of using only rectangular building block [47]. The cylindrical and spherical building block can complement the rectangular building block to provide a more rounded surface on 3D model.

We have noticed jagged edges in our generic and subsequent 3D models. On investigation we found that such jagged edges have been reported on 3D models developed in Blender [48], [49]. This problem is inherent in the Blender software because the image data (pixel value) of 3D model is not accurately calculated when the 3D model is rendered in Blender [50]. The solution to minimize the jagged effect is to use the anti-aliasing option in Blender. We have done this for our generic and new 3D models to reduce the jagged edges but we are not able to completely eliminate the jaggedness. We are currently exploring the use of WebGL programming [51] for a solution to this issue.

In this generic 3D model, the Cartesian coordinates are determined manually from the plot of each point primitive on the Cartesian graph paper (Fig. 2). However, this is a tedious and error prone process especially when the numbers of point primitives assigned are increased at sites of high morphological variation. It might be necessary to improve the method for determining the Cartesian coordinates by using mathematical formula such as the parametric formula of Non-Uniform Rational B-Spline surface (NURBS) to approximate the different degree of curvature of shape found in a natural object [52].

### Usefulness of the newly developed deformable generic 3D anchor model

The derived 3D shapes for the 9 monogenean species indicate that this newly developed deformable 3D model will allow researchers without any prior knowledge of 3D modelling tool to quickly form different desired 3D shapes without going through the tedious 3D modelling process. We have also shown that the deformable generic 3D model created based on a dactylogyridean anchor could be deformed by direct manipulation method into the shape of the hook-like sclerite of a hexabothriid species (see Fig. 9). This shows the usefulness and versatility of our final deformable generic 3D anchor model and also the usefulness of the direct manipulation method which allow us to very rapidly produce a new 3D shape without any problem.

### Application of 3D models in Biology

Although biological organisms are three dimensional in nature, their images (line drawings and photographs) particularly in taxonomy are two dimensional and thus provide an incomplete visualisation of their structural morphology. The development of 3D model would allow us to study and understand the structural and functional morphology as well as the spatial relationships of diagnostic features of organisms. For example, Teo et al. [12] have shown that the rotated views of the whole 3D haptor of a monogenean as an entity allows for the visualisation of the spatial relationship of the diagnostic haptoral hard-parts within the haptor. Rotations of the 3D models have shown that variations in shape could be caused by the orientation of the organism during preservation as indicated by the different anchor shape generated by rotating the 3D anchors through 360° (Fig. 10). This shows that the way the diagnostic parts are oriented during collection and preservation process can affect the shape and morphometric measurements of the specimens and subsequently their taxonomic identification. This supports the need to flatten the worms in such a way as to have a horizontally disposed anchor (lateral view), which gives the best view for measurement and taxonomic comparison [25]. There are instances where specimens belonging to the same species are considered different based on variations in anchor shape caused by orientation (Lim, personal observation). By comparing the shapes of a 3D anchor of a monogenean species in different rotational orientation we can determine whether the anchors belong to the same or different species. It should also be noted that the 3D models have the advantage of being more versatile and the researchers can interactively manipulate and orientate the different constructed 3D parts for a better understanding of how the different structures function [12].

As already briefly noted, members of the 3 subclasses of monogeneans (Polystomatoinea, Oligonchoinea and Polyonchoinea) have great diversity of attachment organs ranging from anchors, bars, marginal hooks, clamps, suckers and haptor (see Introduction). The final generic 3D anchor model can be used to derive 3D shapes of anchors from the different groups of monogeneans (See Fig. 8 & Fig. 9). The method used to produce the present deformable generic 3D anchor can also be used to produce generic 3D models for different hard parts of monogeneans such as marginal hook, bars, clamps and suckers and male and female copulatory organs as well as hooks of trypanorhynch, other cestodes and acanthocephalans and any other organisms. The ultimate aim of this 3D project is to develop a complete whole generic 3D monogenean model by assembling 3D models of the different organs into a 3D outline of a monogenean which can be simulated. To do this it is necessary to develop 3D shapes for the different parts which can be assembled to form a 3D monogenean. Once the 3D parts of the sclerotised hard parts of monogenean (anchors, bars, marginal hooks, squamodisc, suckers, clamps, tegumental spines and scales, male copulatory organ and female organs) as well as soft anatomical structures of male and female reproductive systems, alimentary systems and secretory systems are available, they can be assembled into a 3D body outline of a monogenean (see [12]). By interactively manipulating the different 3D parts we will be able to study the positions and functional morphology of the different organs of the monogeneans particularly the different attachment mechanisms effected by the various haptoral sclerites and structures.

## Supporting Information

Table S1 **Summary of the review on the development of digital 3D Models by different authors.**
(DOC)

Table S2 **Cartesian coordinates X, Y & Z for each vertex on the preliminary generic 3D anchor (manually derived from Cartesian graph paper).**
(DOC)

Table S3 **Cartesian coordinates X, Y & Z for each vertex on the final generic 3D anchor (after optimization of number of point primitives).**
(DOC)

Table S4 **Cartesian coordinates X, Y & Z for each vertex on the 3D anchor of** ***Datylogyrus vastator*** **(manually derived from Cartesian graph paper).**
(DOC)

Table S5 **Cartesian coordinates X, Y & Z for each vertex on the 3D anchor of** ***Dactylogyrus primarius*** **(derived from Transform Properties Window in Blender).**
(DOC)

Table S6 **Cartesian coordinates X, Y & Z for each vertex on the 3D anchor of** ***Pellucidhaptor merus*** **(derived from Transform Properties Window in Blender).**
(DOC)

Table S7 **Cartesian coordinates X, Y & Z for each vertex on the 3D anchor of** ***Dactylogyrus falcatus*** **(derived from Transform Properties Window in Blender).**
(DOC)

Table S8 **Cartesian coordinates X, Y & Z for each vertex on the 3D anchor of** ***Dactylogyrus vastator*** **(derived from Transform Properties Window in Blender).**
(DOC)

Table S9 **Cartesian coordinates X, Y & Z for each vertex on the 3D anchor of** ***Dactylogyrus pterocleidus*** **(derived from Transform Properties Window in Blender).**
(DOC)

Table S10 **Cartesian coordinates X, Y & Z for each vertex on the 3D anchor of** ***Dactylogyrus falciunguis*** **(derived from Transform Properties Window in Blender).**
(DOC)

Table S11 **Cartesian coordinates X, Y & Z for each vertex on the 3D anchor of** ***Chauhanellus auriculatum*** **(derived from Transform Properties Window in Blender).**
(DOC)

Table S12 **Cartesian coordinates X, Y & Z for each vertex on the 3D anchor of** ***Chauhanellus caelatus*** **(derived from Transform Properties Window in Blender).**
(DOC)

Table S13 **Cartesian coordinates X, Y & Z for each vertex on the 3D anchor of** ***Squalonchocotyle mitsukurii*** **(derived from Transform Properties Window in Blender).**
(DOC)

Text S1 **Mathematica codes written to generate the 3D wireframe model for the preliminary generic 3D anchor.**
(NB) Click here for additional data file.

Text S2 **Python script written (in Blender) to generate the 3D wireframe for the preliminary generic 3D model of anchor.**
(PY) Click here for additional data file.

Text S3 **Python script written (in Blender) to generate 3D rectangle as the building blocks for the preliminary generic 3D model.**
(PY) Click here for additional data file.

Text S4 **Python script written (in Blender) to smoothen the preliminary generic 3D model using Catmull-Clark subdivision surface modifier.**
(PY) Click here for additional data file.

Text S5 **Python script written (in Blender) to generate the final deformable generic 3D model of anchor.**
(PY) Click here for additional data file.

Text S6 **Python script written (in Blender) to generate the 3D anchor of** ***Dactylogyrus vastator*** **by changing parameter of Cartesian coordinate in Text S4.**
(PY) Click here for additional data file.
