# Supplementary material for: A Deformable Generic 3D Model of Haptoral Anchor of Monogenean
Source: PLoS One. 2013 Oct 28;8(10):e77650. doi: 10.1371/journal.pone.0077650 (PMC3810373; doi:10.1371/journal.pone.0077650)
Supplement: Table S1 — Summary of the review on the development of digital 3D Models by different authors. (DOC) [file pone.0077650.s001.doc]

**Table S1.** Summary of the review on the development of digital 3D Models by different authors.

| **Types of 3D models (objectives)** | **Target Objects** | **Templates** | **Methods for developing 3D models** | **Authorities (sources)** |
| --- | --- | --- | --- | --- |
| 3D digital model of skull and brain (converted to stereolithograph model by 3D printing) | Human skull and brain | CT image of human skull  MRI image of living human brain | CT scan image of skull and MRI image of brain in ANALYZE format reconstructed to 3D virtual model in vtk format using 3dSlicer and converted to VRML format using MayaVi and finally converted to stereolithograph model through 3D printing using Zcorp Z406 3D printer. | [11] |
| Static 3D model of bee (3D visualisation) | Honey bee | Images of serial sections of honey bee neck | 1. Serial sections of honey bee neck stack to create 3D model using AMIRA 3.1. 2. 3D model smoothened, simplified and corrected for artefact using Silo 2.1. | [6] |
| Static 3D models of arthropods (3D visualisation) | Insects (e.g. ants) & arachnids | 2D images of insects (photographs) | 1. Using primitive shape (spheres, cylinders, boxes) & transformation tools (bending, extruding) in Autodesk 3ds Max, LightWave 2. 3D scanners | [9] |
| Static 3D model of dinosaur (construct body mass) | Dinosaur | Skeleton of dinosaur | 1. Light Detection & Range (LiDAR) imaging technique to digitalize skeletons of dinosaurs into several 3D point clouds. 2. Using Polyworks (software) to merge point clouds into single 3D skeletal model. 3. 3D model refined using RiSCAN PRO to remove unwanted or overlapping points. 4. Autodesk Maya used to construct body outlines around the digital 3D skeletal models. | [10] |
| Static 3D models of haptoral parts of monogenean (3D visualisation & manipulation) | Monogenean haptoral parts (Anchors, bars, marginal hooks) | 2D illustrations of haptoral hard parts of monogeneans | Using primitive shape (cylinders and plane) & transformation tools (bending, extruding, rotating) in Autodesk 3ds Max. | [12] |
| Static 3D models of roof of building (3D visualisation & spatial analysis) | Architecture building | 2D aerial image of a building | 1. **Point primitives** assigned on 2D aerial image of building to represent topological identity of roof. 2. Point primitives connected to generate & group line segments to form a set of 3D polyhedron to construct 3D roof surface. | [27] |
| 3D model of human foot by deforming existing 3D model  (footwear design) | Human foot | 3D model of human foot from 3D foot scanner (INFOOT 3D )  Captured human foot images (target) | 1. Using existing vertices in 3D foot model from scanner 2. **Reflective markers** assigned on target image of human foot. 3. Each Reflective marker on target foot is matched to a set of corresponding vertices on scanned 3D foot model causing displacements of these vertices resulting in 3D model of target foot. | [37] |
| 3D human body model by deforming existing 3D human body model | Human body | Existing 3D model of human body  2D illustration of human body (target image) | 1. Using vertices on existing 3D model 2. **Contour points** assigned on illustration of human body 3. Existing 3D model is deformed by aligning each vertices to the corresponding contour points on 2D illustration forming new 3D model. | [38] |
| Deformable 3D car model using existing generic 3D car model | Car | Existing 3D models of car (source not known)  2D sketch of car (target image) | 1. Fiducial nodes assigned on 3D car model (transforming it into deformable 3D model) 2. Fiducial point assigned on 2D sketch of car. 3. Fiducial nodes of deformable 3D model aligned with fiducial point on 2D sketch to deform 3D model into new shape of 2D sketch | [39] |
| Deformable 3D human face model using existing generic 3D model (portrait-based product development) | Human face | Existing 3D model of human head (Source not known)  2D photographs of frontal and profile views of human face (Target images) | 1. Landmarks points assigned on existing 3D model (transforming it into deformable 3D model) 2. Landmarks points assigned on 2D images 3. Landmarks points on deformable 3D model matched to landmarks points on 2D images 4. Local deformation optimization on non-feature facial regions (eye, nose, mouth) by moving vertices on 3D model following concave-convex texture on 2D images to obtain more realistic 3D face model. | [35] |
| Deformable 3D model using existing 3D ant models | Ants | Existing generic 3D models of ants in VRML format <http://home.comcast.net./~sharov/3d/3dinsect.html>  2D photographs of ants (target images) | 1. Converting existing generic 3D models of ants   from VRML format to 3ds format in Autodesk Max.   1. Selecting control vertices from existing vertices in 3D model creating deformable 3D model 2. Deformable 3D model overlaid on 2D photograph of ants and control vertices on deformable 3D model displaced to match the sketch point of target image. | [33] |
