# Supplementary material for: A Deformable Generic 3D Model of Haptoral Anchor of Monogenean
Source: PLoS One. 2013 Oct 28;8(10):e77650. doi: 10.1371/journal.pone.0077650 (PMC3810373; doi:10.1371/journal.pone.0077650)
Supplement: Table S3 — Cartesian coordinates X, Y & Z for each vertex on the final generic 3D anchor (after optimization of number of point primitives). (DOC) [file pone.0077650.s003.doc]

**Table S3. Cartesian coordinates X, Y & Z for each vertex on the final generic 3D anchor (after optimization of number of point primitives).**

| Set | Vertices | Coordinates-X | Coordinates-Y | Coordinates-Z |
| --- | --- | --- | --- | --- |
| 1 | 1 | -1.2 | 0 | 9.9 |
|  | 2 | -1 | 0 | 10.1 |
|  | 3 | -1 | -0.5 | 10.1 |
|  | 4 | -1.2 | -0.5 | 9.9 |
| 2 | 5 | -1.13 | 0 | 9.6 |
|  | 6 | -0.75 | 0 | 9.8 |
|  | 7 | -0.75 | -0.5 | 9.8 |
|  | 8 | -1.13 | -0.5 | 9.6 |
| 3 | 9 | -0.95 | 0.1 | 9.2 |
|  | 10 | -0.5 | 0.1 | 9.4 |
|  | 11 | -0.5 | -0.6 | 9.4 |
|  | 12 | -0.95 | -0.6 | 9.2 |
| 4 | 13 | -0.85 | 0.1 | 8.8 |
|  | 14 | -0.3 | 0.1 | 9 |
|  | 15 | -0.3 | -0.6 | 9 |
|  | 16 | -0.85 | -0.6 | 8.8 |
| 5 | 17 | -0.67 | 0.15 | 8.3 |
|  | 18 | -0.05 | 0.15 | 8.5 |
|  | 19 | -0.05 | -0.65 | 8.5 |
|  | 20 | -0.67 | -0.65 | 8.3 |
| 6 | 21 | -0.55 | 0.2 | 7.8 |
|  | 22 | 0.2 | 0.2 | 8 |
|  | 23 | 0.2 | -0.7 | 8 |
|  | 24 | -0.55 | -0.7 | 7.8 |
| 7 | 25 | -0.4 | 0.25 | 7.2 |
|  | 26 | 0.55 | 0.25 | 7.4 |
|  | 27 | 0.55 | -0.75 | 7.4 |
|  | 28 | -0.4 | -0.75 | 7.2 |
| 8 | 29 | -0.5 | 0.4 | 6.5 |
|  | 30 | 0.95 | 0.4 | 6.8 |
|  | 31 | 0.95 | -0.9 | 6.8 |
|  | 32 | -0.5 | -0.9 | 6.5 |
| 9 | 33 | -0.4 | 0.5 | 5.7 |
|  | 34 | 1.4 | 0.5 | 6.05 |
|  | 35 | 1.4 | -1 | 6.05 |
|  | 36 | -0.4 | -1 | 5.7 |
| 10 | 37 | -0.3 | 0.5 | 5.3 |
|  | 38 | 1.4 | 0.5 | 5.6 |
|  | 39 | 1.4 | -1 | 5.6 |
|  | 40 | -0.3 | -1 | 5.3 |
| 11 | 41 | 0 | 0.4 | 4.6 |
|  | 42 | 1.15 | 0.4 | 4.8 |
|  | 43 | 1.15 | -0.9 | 4.8 |
|  | 44 | 0 | -0.9 | 4.6 |
| 12 | 45 | 0.05 | 0.4 | 4 |
|  | 46 | 1 | 0.4 | 4 |
|  | 47 | 1 | -0.9 | 4 |
|  | 48 | 0.05 | -0.9 | 4 |
| 13 | 49 | 0.1 | 0.2 | 3.2 |
|  | 50 | 0.9 | 0.2 | 3.2 |
|  | 51 | 0.9 | -0.7 | 3.2 |
|  | 52 | 0.1 | -0.7 | 3.2 |
| 14 | 53 | 0.1 | 0.2 | 2.5 |
|  | 54 | 0.8 | 0.2 | 2.5 |
|  | 55 | 0.8 | -0.7 | 2.5 |
|  | 56 | 0.1 | -0.7 | 2.5 |
| 15 | 57 | -0.1 | 0.1 | 1.9 |
|  | 58 | 0.6 | 0.1 | 1.9 |
|  | 59 | 0.6 | -0.6 | 1.9 |
|  | 60 | -0.1 | -0.6 | 1.9 |
| 16 | 61 | -0.4 | 0.1 | 1.2 |
|  | 62 | 0.3 | 0.1 | 1.1 |
|  | 63 | 0.3 | -0.6 | 1.1 |
|  | 64 | -0.4 | -0.6 | 1.2 |
| 17 | 65 | -0.7 | 0.1 | 0.8 |
|  | 66 | -0.2 | 0.1 | 0.4 |
|  | 67 | -0.2 | -0.6 | 0.4 |
|  | 68 | -0.7 | -0.6 | 0.8 |
| 18 | 69 | -1.0 | 0 | 0.5 |
|  | 70 | -1 | 0 | 0 |
|  | 71 | -1 | -0.5 | 0 |
|  | 72 | -1 | -0.5 | 0.5 |
| 19 | 73 | -1.5 | 0 | 0.9 |
|  | 74 | -1.5 | 0 | 0.3 |
|  | 75 | -1.5 | -0.5 | 0.3 |
|  | 76 | -1.5 | -0.5 | 0.9 |
| 20 | 77 | -2 | 0 | 1.55 |
|  | 78 | -2 | 0 | 0.95 |
|  | 79 | -2 | -0.5 | 0.95 |
|  | 80 | -2 | -0.5 | 1.55 |
| 21 | 81 | -2.4 | 0 | 2.1 |
|  | 82 | -2.4 | 0 | 1.5 |
|  | 83 | -2.4 | -0.5 | 1.5 |
|  | 84 | -2.4 | -0.5 | 2.1 |
| 22 | 85 | -2.8 | -0.1 | 2.7 |
|  | 86 | -2.8 | -0.1 | 2.3 |
|  | 87 | -2.8 | -0.4 | 2.3 |
|  | 88 | -2.8 | -0.4 | 2.7 |
| 23 | 89 | -3.4 | -0.2 | 3.5 |
|  | 90 | -3.4 | -0.2 | 3.3 |
|  | 91 | -3.4 | -0.3 | 3.3 |
|  | 92 | -3.3 | -0.3 | 3.5 |
| 24 | 93 | -1.15 | -0.15 | 9.6 |
|  | 94 | -1.15 | -0.35 | 9.6 |
|  | 95 | -1 | -0.35 | 9.2 |
|  | 96 | -1 | -0.15 | 9.2 |
| 25 | 97 | -0.85 | -0.15 | 8.8 |
|  | 98 | -0.85 | -0.35 | 8.8 |
|  | 99 | -0.67 | -0.35 | 8.3 |
|  | 100 | -0.67 | -0.15 | 8.3 |
| 26 | 101 | 0.95 | -0.2 | 6.8 |
|  | 102 | 0.95 | -0.3 | 6.8 |
|  | 103 | 1.4 | -0.3 | 6.05 |
|  | 104 | 1.4 | -0.2 | 6.05 |
| 27 | 105 | -0.45 | -0.2 | 1.1 |
|  | 106 | -0.5 | -0.3 | 1.05 |
|  | 107 | -0.5 | -0.2 | 1.05 |
|  | 108 | -2.1 | -0.2 | 1.7 |
| 28 | 109 | -2.1 | -0.3 | 1.7 |
|  | 110 | -2.2 | -0.3 | 1.85 |
|  | 111 | -2.2 | -0.2 | 1.85 |
|  | 112 | -0.4 | -0.15 | 7.2 |
| 29 | 113 | -0.5 | -0.35 | 6.5 |
|  | 114 | -0.5 | -0.15 | 6.5 |
|  | 115 | -0.45 | -0.22 | 1.1 |
|  | 116 | -0.45 | -0.28 | 1.1 |
| 30 | 117 | -0.5 | -0.28 | 1.05 |
|  | 118 | -0.5 | -0.22 | 1.05 |
|  | 119 | -2.1 | -0.22 | 1.7 |
|  | 120 | -2.1 | -0.28 | 1.7 |
| 31 | 121 | -2.2 | -0.28 | 1.85 |
|  | 122 | -2.2 | -0.22 | 1.85 |
|  | 123 | -2.1 | -0.24 | 1.7 |
|  | 124 | -2.1 | -0.26 | 1.7 |
| 32 | 125 | -2.2 | -0.26 | 1.85 |
|  | 126 | -2.2 | -0.24 | 1.85 |
|  | 127 | -0.5 | -0.24 | 1.05 |
|  | 128 | -0.5 | -0.26 | 1.05 |
| 33 | 129 | -0.45 | -0.26 | 1.1 |
|  | 130 | -0.45 | -0.24 | 1.1 |
|  | 131 | 0.9 | -0.2 | 3.2 |
|  | 132 | 0.7 | -0.2 | 2.5 |
