# Supplementary material for: A Deformable Generic 3D Model of Haptoral Anchor of Monogenean
Source: PLoS One. 2013 Oct 28;8(10):e77650. doi: 10.1371/journal.pone.0077650 (PMC3810373; doi:10.1371/journal.pone.0077650)
Supplement: Table S4 — Cartesian coordinates X, Y & Z for each vertex on the 3D anchor of Datylogyrus vastator (manually derived from Cartesian graph paper). (DOC) [file pone.0077650.s004.doc]

**Table S4. Cartesian coordinates X, Y & Z for each vertex on the 3D anchor of *Datylogyrus vastator* (manually derived from Cartesian graph paper).**

| Set | Vertices | Coordinates-X | Coordinates-Y | Coordinates-Z |
| --- | --- | --- | --- | --- |
| 1 | 1 | -1.86 | 0.34 | 10.21 |
|  | 2 | 1.21 | 0.34 | 9.82 |
|  | 3 | 1.21 | -0.84 | 9.82 |
|  | 4 | -1.86 | -0.84 | 10.22 |
| 2 | 5 | -1.58 | 0.34 | 9.60 |
|  | 6 | 1.48 | 0.34 | 9.50 |
|  | 7 | 1.48 | -0.84 | 9.50 |
|  | 8 | -1.58 | -0.84 | 9.60 |
| 3 | 9 | -1.61 | 0.61 | 9.20 |
|  | 10 | 1.72 | 0.58 | 9.13 |
|  | 11 | 1.72 | -1.08 | 9.13 |
|  | 12 | -1.61 | -1.11 | 9.20 |
| 4 | 13 | -1.50 | 0.67 | 8.80 |
|  | 14 | 1.70 | 0.67 | 8.75 |
|  | 15 | 1.70 | -1.17 | 8.75 |
|  | 16 | -1.50 | -1.17 | 8.80 |
| 5 | 17 | -1.46 | 0.70 | 8.30 |
|  | 18 | 1.74 | 0.70 | 8.34 |
|  | 19 | 1.74 | -1.20 | 8.34 |
|  | 20 | -1.46 | -1.20 | 8.30 |
| 6 | 21 | -1.34 | 0.79 | 7.80 |
|  | 22 | 1.69 | 0.79 | 8 |
|  | 23 | 1.69 | -1.29 | 8 |
|  | 24 | -1.34 | -1.29 | 7.80 |
| 7 | 25 | -1.19 | 0.72 | 7.20 |
|  | 26 | 1.73 | 0.90 | 7.40 |
|  | 27 | 1.73 | -1.40 | 7.40 |
|  | 28 | -1.19 | -1.22 | 7.20 |
| 8 | 29 | -0.90 | 0.81 | 6.50 |
|  | 30 | 1.63 | 0.82 | 6.80 |
|  | 31 | 1.63 | -1.32 | 6.80 |
|  | 32 | -0.90 | -1.32 | 6.50 |
| 9 | 33 | -0.41 | 0.50 | 5.70 |
|  | 34 | 1.53 | 0.50 | 6.05 |
|  | 35 | 1.53 | -1 | 6.05 |
|  | 36 | -0.41 | -1 | 5.70 |
| 10 | 37 | -0.11 | 0.50 | 5.30 |
|  | 38 | 1.53 | 0.50 | 5.60 |
|  | 39 | 1.53 | -1 | 5.60 |
|  | 40 | -0.11 | -1 | 5.30 |
| 11 | 41 | 0.12 | 0.40 | 4.60 |
|  | 42 | 1.50 | 0.40 | 4.80 |
|  | 43 | 1.50 | -0.90 | 4.80 |
|  | 44 | 0.12 | -0.90 | 4.60 |
| 12 | 45 | 0.17 | 0.40 | 4 |
|  | 46 | 1.35 | 0.40 | 4 |
|  | 47 | 1.35 | -0.90 | 4 |
|  | 48 | 0.17 | -0.90 | 4 |
| 13 | 49 | 0.30 | 0.20 | 3.20 |
|  | 50 | 1.23 | 0.20 | 3.20 |
|  | 51 | 1.23 | -0.70 | 3.20 |
|  | 52 | 0.30 | -0.70 | 3.20 |
| 14 | 53 | 0.30 | 0.20 | 2.50 |
|  | 54 | 1.13 | 0.20 | 2.50 |
|  | 55 | 1.13 | -0.70 | 2.50 |
|  | 56 | 0.30 | -0.70 | 2.50 |
| 15 | 57 | 0.24 | 0.10 | 1.90 |
|  | 58 | 1.07 | 0.10 | 1.90 |
|  | 59 | 1.07 | -0.60 | 1.90 |
|  | 60 | 0.24 | -0.60 | 1.90 |
| 16 | 61 | 0.10 | 0.10 | 1.11 |
|  | 62 | 0.80 | 0.10 | 1.01 |
|  | 63 | 0.80 | -0.60 | 1.01 |
|  | 64 | 0.10 | -0.60 | 1.11 |
| 17 | 65 | -0.04 | 0.10 | 0.45 |
|  | 66 | 0.67 | 0.10 | 0.26 |
|  | 67 | 0.67 | -0.60 | 0.26 |
|  | 68 | -0.04 | -0.60 | 0.45 |
| 18 | 69 | -0.24 | 0 | -0.13 |
|  | 70 | 0.40 | 0 | -0.45 |
|  | 71 | 0.40 | -0.50 | -0.46 |
|  | 72 | -0.24 | -0.50 | -0.13 |
| 19 | 73 | -0.53 | 0 | -0.93 |
|  | 74 | 0.14 | 0 | -1.23 |
|  | 75 | 0.14 | -0.50 | -1.23 |
|  | 76 | -0.53 | -0.50 | -0.93 |
| 20 | 77 | -0.78 | 0 | -1.47 |
|  | 78 | -0.37 | 0 | -2.26 |
|  | 79 | -0.37 | -0.50 | -2.26 |
|  | 80 | -0.78 | -0.50 | -1.47 |
| 21 | 81 | -1.54 | 0 | -1.69 |
|  | 82 | -1.48 | 0 | -2.29 |
|  | 83 | -1.48 | -0.50 | -2.29 |
|  | 84 | -1.54 | -0.50 | -1.69 |
| 22 | 85 | -2.12 | -0.10 | -1.57 |
|  | 86 | -2.08 | -0.10 | -1.96 |
|  | 87 | -2.08 | -0.40 | -1.96 |
|  | 88 | -2.12 | -0.40 | -1.57 |
| 23 | 89 | -3.23 | -0.20 | -0.93 |
|  | 90 | -3.21 | -0.20 | -1.13 |
|  | 91 | -3.21 | -0.30 | -1.13 |
|  | 92 | -3.13 | -0.30 | -0.92 |
