# Supplementary material for: A Deformable Generic 3D Model of Haptoral Anchor of Monogenean
Source: PLoS One. 2013 Oct 28;8(10):e77650. doi: 10.1371/journal.pone.0077650 (PMC3810373; doi:10.1371/journal.pone.0077650)
Supplement: Table S5 — Cartesian coordinates X, Y & Z for each vertex on the 3D anchor of Dactylogyrus primarius (derived from Transform Properties Window in Blender). (DOC) [file pone.0077650.s005.doc]

**Table S5. Cartesian coordinates X, Y & Z for each vertex on the 3D anchor of *Dactylogyrus primarius* (derived from Transform Properties Window in Blender).**

| Set | Vertices | Coordinates-X | Coordinates-Y | Coordinates-Z |
| --- | --- | --- | --- | --- |
| 1 | 1 | -2.04 | 0.17 | 5.99 |
|  | 2 | -2.02 | 0.17 | 6.73 |
|  | 3 | -2.02 | -0.67 | 6.73 |
|  | 4 | -2.04 | -0.67 | 5.99 |
| 2 | 5 | -1.45 | 0.17 | 5.59 |
|  | 6 | -1.64 | 0.17 | 6.91 |
|  | 7 | -1.64 | -0.67 | 6.91 |
|  | 8 | -1.45 | -0.67 | 5.89 |
| 3 | 9 | -1.17 | 0.34 | 5.95 |
|  | 10 | -1.39 | 0.34 | 7.06 |
|  | 11 | -1.39 | -0.84 | 7.06 |
|  | 12 | -1.17 | -0.84 | 5.95 |
| 4 | 13 | -0.83 | 0.36 | 6.01 |
|  | 14 | -0.90 | 0.34 | 7.36 |
|  | 15 | -0.90 | -0.84 | 7.36 |
|  | 16 | -0.83 | -0.86 | 6.01 |
| 5 | 17 | -0.68 | 0.35 | 6.08 |
|  | 18 | -0.63 | 0.35 | 7.67 |
|  | 19 | -0.63 | -0.85 | 7.67 |
|  | 20 | -0.68 | -0.85 | 6.08 |
| 6 | 21 | -0.44 | 0.36 | 6.18 |
|  | 22 | -0.38 | 0.36 | 8.03 |
|  | 23 | -0.38 | -0.86 | 8.03 |
|  | 24 | -0.44 | -0.86 | 6.18 |
| 7 | 25 | -0.12 | 0.43 | 6.27 |
|  | 26 | -0.16 | 0.43 | 8.47 |
|  | 27 | -0.16 | -0.93 | 8.47 |
|  | 28 | -0.12 | -0.93 | 6.27 |
| 8 | 29 | 0.17 | 0.40 | 6.15 |
|  | 30 | 0.18 | 0.40 | 9.36 |
|  | 31 | 0.18 | -0.90 | 9.36 |
|  | 32 | 0.17 | -0.90 | 6.15 |
| 9 | 33 | 0.38 | 0.50 | 6.23 |
|  | 34 | 1.58 | 0.50 | 8.62 |
|  | 35 | 1.58 | -1 | 8.62 |
|  | 36 | 0.38 | -1 | 6.23 |
| 10 | 37 | 0.55 | 0.50 | 5.30 |
|  | 38 | 1.59 | 0.50 | 6.68 |
|  | 39 | 1.59 | -1 | 6.68 |
|  | 40 | 0.55 | -1 | 5.30 |
| 11 | 41 | 0.84 | 0.40 | 4.60 |
|  | 42 | 1.78 | 0.40 | 4.80 |
|  | 43 | 1.78 | -0.90 | 4.80 |
|  | 44 | 0.84 | -0.90 | 4.60 |
| 12 | 45 | 0.89 | 0.40 | 4 |
|  | 46 | 1.84 | 0.40 | 4 |
|  | 47 | 1.84 | -0.90 | 4 |
|  | 48 | 0.89 | -0.90 | 4 |
| 13 | 49 | 0.95 | 0.29 | 3.20 |
|  | 50 | 1.88 | 0.29 | 3.20 |
|  | 51 | 1.88 | -0.79 | 3.20 |
|  | 52 | 0.95 | -0.79 | 3.20 |
| 14 | 53 | 0.76 | 0.29 | 2.50 |
|  | 54 | 1.65 | 0.29 | 2.50 |
|  | 55 | 1.65 | -0.79 | 2.50 |
|  | 56 | 0.76 | -0.79 | 2.50 |
| 15 | 57 | 0.73 | 0.17 | 1.90 |
|  | 58 | 1.53 | 0.17 | 1.90 |
|  | 59 | 1.53 | -0.67 | 1.90 |
|  | 60 | 0.73 | -0.67 | 1.90 |
| 16 | 61 | 0.58 | 0.17 | 1.20 |
|  | 62 | 1.29 | 0.17 | 1.10 |
|  | 63 | 1.29 | -0.67 | 1.10 |
|  | 64 | 0.58 | -0.67 | 1.20 |
| 17 | 65 | 0.38 | 0.17 | 0.59 |
|  | 66 | 0.94 | 0.17 | 0.06 |
|  | 67 | 0.94 | -0.67 | 0.06 |
|  | 68 | 0.38 | -0.68 | 0.59 |
| 18 | 69 | 0.12 | 0.05 | 0.05 |
|  | 70 | 0.73 | 0.05 | -0.59 |
|  | 71 | 0.73 | -0.55 | -0.59 |
|  | 72 | 0.12 | -0.55 | 0.05 |
| 19 | 73 | -0.14 | 0.05 | -0.93 |
|  | 74 | -0.20 | 0.05 | -1.76 |
|  | 75 | -0.20 | -0.55 | -1.76 |
|  | 76 | -0.14 | -0.55 | -0.92 |
| 20 | 77 | -1.14 | 0.05 | -0.45 |
|  | 78 | -1.14 | 0.05 | -1.10 |
|  | 79 | -1.14 | -0.55 | -1.10 |
|  | 80 | -1.14 | -0.55 | -0.45 |
| 21 | 81 | -1.54 | 0.05 | -0.29 |
|  | 82 | -1.64 | 0.05 | -0.73 |
|  | 83 | -1.64 | -0.55 | -0.73 |
|  | 84 | -1.54 | -0.55 | -0.29 |
| 22 | 85 | -1.94 | -0.07 | -0.22 |
|  | 86 | -1.94 | -0.07 | -0.62 |
|  | 87 | -1.94 | -0.43 | -0.62 |
|  | 88 | -1.94 | -0.43 | -0.22 |
| 23 | 89 | -3.08 | -0.15 | -0.07 |
|  | 90 | -3.08 | -0.15 | -0.27 |
|  | 91 | -3.08 | -0.35 | -0.27 |
|  | 92 | -2.98 | -0.35 | -0.07 |
| 24 | 93 | 0.21 | 0.24 | 10.84 |
|  | 94 | 0.21 | -0.74 | 10.84 |
|  | 95 | 2.06 | -0.74 | 9.91 |
|  | 96 | 2.06 | 0.24 | 9.91 |
