# Supplementary material for: A Deformable Generic 3D Model of Haptoral Anchor of Monogenean
Source: PLoS One. 2013 Oct 28;8(10):e77650. doi: 10.1371/journal.pone.0077650 (PMC3810373; doi:10.1371/journal.pone.0077650)
Supplement: Table S6 — Cartesian coordinates X, Y & Z for each vertex on the 3D anchor of Pellucidhaptor merus (derived from Transform Properties Window in Blender). (DOC) [file pone.0077650.s006.doc]

**Table S6. Cartesian coordinates X, Y & Z for each vertex on the 3D anchor of *Pellucidhaptor merus* (derived from Transform Properties Window in Blender).**

| Set | Vertices | Coordinates-X | Coordinates-Y | Coordinates-Z |
| --- | --- | --- | --- | --- |
| 1 | 1 | -2.48 | 0.15 | 11.53 |
|  | 2 | -1.65 | 0.15 | 11.63 |
|  | 3 | -1.65 | -0.65 | 11.63 |
|  | 4 | -2.48 | -0.65 | 11.53 |
| 2 | 5 | -2.42 | 0.15 | 11.08 |
|  | 6 | -1.35 | 0.15 | 11.28 |
|  | 7 | -1.35 | -0.65 | 11.28 |
|  | 8 | -2.42 | -0.65 | 11.08 |
| 3 | 9 | -2.19 | 0.30 | 10.68 |
|  | 10 | -1.03 | 0.30 | 10.88 |
|  | 11 | -1.04 | -0.80 | 10.88 |
|  | 12 | -2.19 | -0.80 | 10.68 |
| 4 | 13 | -2.13 | 0.30 | 10.28 |
|  | 14 | -0.84 | 0.30 | 10.48 |
|  | 15 | -0.85 | -0.80 | 10.48 |
|  | 16 | -2.13 | -0.80 | 10.28 |
| 5 | 17 | -2.01 | 0.38 | 9.78 |
|  | 18 | -0.68 | 0.38 | 9.98 |
|  | 19 | -0.68 | -0.88 | 9.98 |
|  | 20 | -2.01 | -0.88 | 9.78 |
| 6 | 21 | -2.16 | 0.46 | 9.28 |
|  | 22 | -0.34 | 0.46 | 9.48 |
|  | 23 | -0.34 | -0.96 | 9.48 |
|  | 24 | -2.16 | -0.96 | 9.28 |
| 7 | 25 | -1.94 | 0.54 | 7.54 |
|  | 26 | -0.08 | 0.54 | 8.89 |
|  | 27 | -0.08 | -1.04 | 8.89 |
|  | 28 | -1.94 | -1.04 | 7.54 |
| 8 | 29 | -0.76 | 0.65 | 6.12 |
|  | 30 | 0.53 | 0.65 | 8.75 |
|  | 31 | 0.53 | -1.15 | 8.75 |
|  | 32 | -0.76 | -1.15 | 6.12 |
| 9 | 33 | 0 | 0.75 | 5.70 |
|  | 34 | 2.09 | 0.56 | 7.99 |
|  | 35 | 2.09 | -1.06 | 7.99 |
|  | 36 | 0 | -1.25 | 5.70 |
| 10 | 37 | 0.70 | 0.50 | 5.30 |
|  | 38 | 2.84 | 0.50 | 5.60 |
|  | 39 | 2.84 | -1 | 5.60 |
|  | 40 | 0.70 | -1 | 5.30 |
| 11 | 41 | 1.25 | 0.40 | 4.60 |
|  | 42 | 2.72 | 0.40 | 4.80 |
|  | 43 | 2.72 | -0.90 | 4.80 |
|  | 44 | 1.25 | -0.90 | 4.60 |
| 12 | 45 | 1.49 | 0.40 | 4 |
|  | 46 | 2.77 | 0.40 | 4 |
|  | 47 | 2.77 | -0.90 | 4 |
|  | 48 | 1.49 | -0.90 | 4 |
| 13 | 49 | 1.78 | 0.20 | 3.20 |
|  | 50 | 2.72 | 0.20 | 3.20 |
|  | 51 | 2.72 | -0.70 | 3.20 |
|  | 52 | 1.78 | -0.70 | 3.20 |
| 14 | 53 | 1.72 | 0.20 | 2.50 |
|  | 54 | 2.47 | 0.20 | 2.30 |
|  | 55 | 2.47 | -0.70 | 2.31 |
|  | 56 | 1.72 | -0.70 | 2.50 |
| 15 | 57 | 1.35 | 0.15 | 1.90 |
|  | 58 | 2.16 | 0.15 | 1.71 |
|  | 59 | 2.16 | -0.65 | 1.71 |
|  | 60 | 1.35 | -0.65 | 1.90 |
| 16 | 61 | 0.56 | 0.15 | 1.20 |
|  | 62 | 1.46 | 0.15 | 0.92 |
|  | 63 | 1.46 | -0.65 | 0.92 |
|  | 64 | 0.56 | -0.65 | 1.20 |
| 17 | 65 | 0.08 | 0.15 | 0.80 |
|  | 66 | 0.58 | 0.15 | 0.21 |
|  | 67 | 0.58 | -0.65 | 0.21 |
|  | 68 | 0.08 | -0.65 | 0.80 |
| 18 | 69 | -0.48 | 0.13 | 0.45 |
|  | 70 | -0.48 | 0.13 | -0.32 |
|  | 71 | -0.48 | -0.63 | -0.32 |
|  | 72 | -0.48 | -0.63 | 0.45 |
| 19 | 73 | -1.53 | 0.13 | 0.05 |
|  | 74 | -1.53 | 0.13 | -0.66 |
|  | 75 | -1.53 | -0.63 | -0.66 |
|  | 76 | -1.53 | -0.63 | 0.05 |
| 20 | 77 | -2.57 | 0.13 | -0.36 |
|  | 78 | -2.57 | 0.13 | -1.04 |
|  | 79 | -2.57 | -0.63 | -1.04 |
|  | 80 | -2.57 | -0.63 | -0.36 |
| 21 | 81 | -4.02 | 0.13 | -0.40 |
|  | 82 | -4.02 | 0.13 | -1 |
|  | 83 | -4.02 | -0.63 | -1 |
|  | 84 | -4.02 | -0.63 | -0.40 |
| 22 | 85 | -4.92 | -0.02 | -0.25 |
|  | 86 | -4.92 | -0.02 | -0.65 |
|  | 87 | -4.92 | -0.48 | -0.65 |
|  | 88 | -4.92 | -0.48 | -0.25 |
| 23 | 89 | -5.40 | -0.17 | 0.58 |
|  | 90 | -5.40 | -0.17 | 0.38 |
|  | 91 | -5.40 | -0.33 | 0.38 |
|  | 92 | -5.30 | -0.33 | 0.58 |
| 24 | 93 | 0.76 | 0.12 | 9.63 |
|  | 94 | 0.76 | -0.62 | 9.63 |
|  | 95 | 1.66 | -0.62 | 9.28 |
|  | 96 | 1.66 | 0.12 | 9.28 |
