# Supplementary material for: A Deformable Generic 3D Model of Haptoral Anchor of Monogenean
Source: PLoS One. 2013 Oct 28;8(10):e77650. doi: 10.1371/journal.pone.0077650 (PMC3810373; doi:10.1371/journal.pone.0077650)
Supplement: Table S7 — Cartesian coordinates X, Y & Z for each vertex on the 3D anchor of Dactylogyrus falcatus (derived from Transform Properties Window in Blender). (DOC) [file pone.0077650.s007.doc]

**Table S7. Cartesian coordinates X, Y & Z for each vertex on the 3D anchor of *Dactylogyrus falcatus* (derived from Transform Properties Window in Blender).**

| Set | Vertices | Coordinates-X | Coordinates-Y | Coordinates-Z |
| --- | --- | --- | --- | --- |
| 1 | 1 | -4.97 | 0.20 | 8.11 |
|  | 2 | -3.46 | 0.20 | 9.01 |
|  | 3 | -3.46 | -0.70 | 9.01 |
|  | 4 | -4.97 | -0.70 | 8.11 |
| 2 | 5 | -3.82 | 0.20 | 7.38 |
|  | 6 | -2.92 | 0.20 | 8.61 |
|  | 7 | -2.92 | -0.70 | 8.61 |
|  | 8 | -3.82 | -0.70 | 7.38 |
| 3 | 9 | -3.40 | 0.36 | 7.17 |
|  | 10 | -2.46 | 0.38 | 8.42 |
|  | 11 | -2.46 | -0.88 | 8.42 |
|  | 12 | -3.40 | -0.86 | 7.17 |
| 4 | 13 | -3.06 | 0.51 | 6.92 |
|  | 14 | -1.93 | 0.38 | 8.06 |
|  | 15 | -1.93 | -0.88 | 8.06 |
|  | 16 | -3.06 | -1.01 | 6.92 |
| 5 | 17 | -2.64 | 0.47 | 6.63 |
|  | 18 | -1.55 | 0.47 | 7.91 |
|  | 19 | -1.55 | -0.97 | 7.91 |
|  | 20 | -2.64 | -0.97 | 6.63 |
| 6 | 21 | -2.25 | 0.41 | 6.21 |
|  | 22 | -1.11 | 0.57 | 7.70 |
|  | 23 | -1.11 | -1.07 | 7.70 |
|  | 24 | -2.25 | -0.91 | 6.21 |
| 7 | 25 | -1.80 | 0.55 | 6.02 |
|  | 26 | -0.66 | 0.62 | 7.40 |
|  | 27 | -0.66 | -1.12 | 7.40 |
|  | 28 | -1.80 | -1.05 | 6.02 |
| 8 | 29 | -1.23 | 0.40 | 5.62 |
|  | 30 | 0.01 | 0.52 | 6.97 |
|  | 31 | 0.01 | -1.02 | 6.97 |
|  | 32 | -1.23 | -0.90 | 5.62 |
| 9 | 33 | -0.82 | 0.50 | 5.31 |
|  | 34 | 1.66 | 0.64 | 6.05 |
|  | 35 | 1.66 | -1.14 | 6.05 |
|  | 36 | -0.82 | -1 | 5.31 |
| 10 | 37 | -0.50 | 0.50 | 4.95 |
|  | 38 | 1.44 | 0.50 | 5.25 |
|  | 39 | 1.44 | -1 | 5.25 |
|  | 40 | -0.50 | -1 | 4.95 |
| 11 | 41 | -0.40 | 0.40 | 4.60 |
|  | 42 | 1.22 | 0.40 | 4.80 |
|  | 43 | 1.22 | -0.90 | 4.80 |
|  | 44 | -0.40 | -0.90 | 4.60 |
| 12 | 45 | -0.35 | 0.40 | 3.52 |
|  | 46 | 0.78 | 0.40 | 3.52 |
|  | 47 | 0.78 | -0.90 | 3.52 |
|  | 48 | -0.35 | -0.90 | 3.52 |
| 13 | 49 | -0.82 | 0.20 | 2.81 |
|  | 50 | 0.29 | 0.20 | 2.52 |
|  | 51 | 0.29 | -0.70 | 2.52 |
|  | 52 | -0.82 | -0.70 | 2.81 |
| 14 | 53 | -1.67 | 0.20 | 2.19 |
|  | 54 | -0.64 | 0.20 | 1.75 |
|  | 55 | -0.64 | -0.70 | 1.75 |
|  | 56 | -1.67 | -0.70 | 2.19 |
| 15 | 57 | -2.07 | 0.10 | 2 |
|  | 58 | -1.84 | 0.10 | 1.23 |
|  | 59 | -1.84 | -0.60 | 1.23 |
|  | 60 | -2.07 | -0.60 | 2 |
| 16 | 61 | -2.61 | 0.10 | 1.85 |
|  | 62 | -2.26 | 0.10 | 1.17 |
|  | 63 | -2.26 | -0.60 | 1.17 |
|  | 64 | -2.61 | -0.60 | 1.85 |
| 17 | 65 | -3.14 | 0.10 | 1.67 |
|  | 66 | -2.73 | 0.10 | 1.10 |
|  | 67 | -2.73 | -0.60 | 1.10 |
|  | 68 | -3.13 | -0.60 | 1.67 |
| 18 | 69 | -3.90 | 0 | 1.20 |
|  | 70 | -3.65 | 0 | 0.58 |
|  | 71 | -3.65 | -0.50 | 0.58 |
|  | 72 | -3.90 | -0.50 | 1.20 |
| 19 | 73 | -4.45 | 0 | 0.96 |
|  | 74 | -4.43 | 0 | 0.43 |
|  | 75 | -4.43 | -0.50 | 0.43 |
|  | 76 | -4.45 | -0.50 | 0.96 |
| 20 | 77 | -4.92 | 0 | 0.82 |
|  | 78 | -4.93 | 0 | 0.30 |
|  | 79 | -4.93 | -0.50 | 0.30 |
|  | 80 | -4.92 | -0.50 | 0.82 |
| 21 | 81 | -5.69 | 0 | 0.63 |
|  | 82 | -5.72 | 0 | 0.24 |
|  | 83 | -5.72 | -0.50 | 0.24 |
|  | 84 | -5.69 | -0.50 | 0.63 |
| 22 | 85 | -6.34 | -0.10 | 0.66 |
|  | 86 | -6.42 | -0.10 | 0.26 |
|  | 87 | -6.42 | -0.40 | 0.26 |
|  | 88 | -6.34 | -0.40 | 0.66 |
| 23 | 89 | -6.62 | -0.20 | 1.21 |
|  | 90 | -6.66 | -0.20 | 1.02 |
|  | 91 | -6.66 | -0.30 | 1.02 |
|  | 92 | -6.52 | -0.30 | 1.19 |
| 24 | 93 | 1.23 | 0.37 | 9.56 |
|  | 94 | 1.23 | -0.87 | 9.56 |
|  | 95 | 3.18 | -0.87 | 8.46 |
|  | 96 | 3.18 | 0.37 | 8.46 |
