# Supplementary material for: A Deformable Generic 3D Model of Haptoral Anchor of Monogenean
Source: PLoS One. 2013 Oct 28;8(10):e77650. doi: 10.1371/journal.pone.0077650 (PMC3810373; doi:10.1371/journal.pone.0077650)
Supplement: Table S8 — Cartesian coordinates X, Y & Z for each vertex on the 3D anchor of Dactylogyrus vastator (derived from Transform Properties Window in Blender). (DOC) [file pone.0077650.s008.doc]

**Table S8. Cartesian coordinates X, Y & Z for each vertex on the 3D anchor of *Dactylogyrus vastator* (derived from Transform Properties Window in Blender).**

| Set | Vertices | Coordinates-X | Coordinates-Y | Coordinates-Z |
| --- | --- | --- | --- | --- |
| 1 | 1 | -2.24 | 0.07 | 10.88 |
|  | 2 | -1.37 | 0.07 | 11.14 |
|  | 3 | -1.37 | -0.57 | 11.14 |
|  | 4 | -2.24 | -0.57 | 10.88 |
| 2 | 5 | -1.98 | 0.14 | 10.39 |
|  | 6 | -0.99 | 0.14 | 10.59 |
|  | 7 | -0.99 | -0.64 | 10.59 |
|  | 8 | -1.98 | -0.64 | 10.39 |
| 3 | 9 | -1.75 | 0.19 | 9.99 |
|  | 10 | -0.67 | 0.19 | 10.19 |
|  | 11 | -0.67 | -0.69 | 10.19 |
|  | 12 | -1.75 | -0.69 | 9.99 |
| 4 | 13 | -1.54 | 0.24 | 9.51 |
|  | 14 | -0.49 | 0.24 | 9.71 |
|  | 15 | -0.49 | -0.74 | 9.71 |
|  | 16 | -1.54 | -0.74 | 9.51 |
| 5 | 17 | -1.46 | 0.26 | 9 |
|  | 18 | -0.16 | 0.26 | 9.21 |
|  | 19 | -0.16 | -0.76 | 9.21 |
|  | 20 | -1.46 | -0.76 | 9 |
| 6 | 21 | -1.30 | 0.32 | 8.47 |
|  | 22 | -0.05 | 0.32 | 8.68 |
|  | 23 | -0.05 | -0.82 | 8.68 |
|  | 24 | -1.31 | -0.82 | 8.47 |
| 7 | 25 | -1.27 | 0.38 | 7.20 |
|  | 26 | 0.19 | 0.38 | 8.20 |
|  | 27 | 0.19 | -0.88 | 8.20 |
|  | 28 | -1.27 | -0.88 | 7.20 |
| 8 | 29 | -1 | 0.40 | 6.50 |
|  | 30 | 0.59 | 0.40 | 7.47 |
|  | 31 | 0.59 | -0.90 | 7.47 |
|  | 32 | -1 | -0.90 | 6.50 |
| 9 | 33 | -0.65 | 0.50 | 5.70 |
|  | 34 | 1.21 | 0.50 | 6.72 |
|  | 35 | 1.21 | -1 | 6.72 |
|  | 36 | -0.65 | -1 | 5.70 |
| 10 | 37 | -0.45 | 0.50 | 5.30 |
|  | 38 | 0.91 | 0.50 | 5.60 |
|  | 39 | 0.91 | -1 | 5.60 |
|  | 40 | -0.45 | -1 | 5.30 |
| 11 | 41 | -0.25 | 0.40 | 4.60 |
|  | 42 | 0.90 | 0.40 | 4.80 |
|  | 43 | 0.90 | -0.90 | 4.80 |
|  | 44 | -0.25 | -0.90 | 4.60-0.10 |
| 12 | 45 | -0.10 | 0.34 | 4.20 |
|  | 46 | 0.85 | 0.34 | 4.20 |
|  | 47 | 0.85 | -0.84 | 4.21 |
|  | 48 | -0.10 | -0.84 | 4.21 |
| 13 | 49 | -0.05 | 0.29 | 3.77 |
|  | 50 | 0.79 | 0.29 | 3.77 |
|  | 51 | 0.79 | -0.79 | 3.77 |
|  | 52 | -0.05 | -0.79 | 3.77 |
| 14 | 53 | -0.05 | 0.29 | 3.26 |
|  | 54 | 0.84 | 0.29 | 3.26 |
|  | 55 | 0.84 | -0.79 | 3.26 |
|  | 56 | -0.05 | -0.79 | 3.26 |
| 15 | 57 | -0.10 | 0.17 | 2.25 |
|  | 58 | 0.73 | 0.17 | 1.90 |
|  | 59 | 0.73 | -0.67 | 1.90 |
|  | 60 | -0.10 | -0.67 | 2.25 |
| 16 | 61 | -0.40 | 0.17 | 1.91 |
|  | 62 | 0.53 | 0.17 | 1.10 |
|  | 63 | 0.53 | -0.67 | 1.10 |
|  | 64 | -0.40 | -0.67 | 1.91 |
| 17 | 65 | -0.59 | 0.17 | 0.43 |
|  | 66 | 0.04 | 0.17 | -0.13 |
|  | 67 | 0.04 | -0.67 | -0.13 |
|  | 68 | -0.59 | -0.67 | 0.43 |
| 18 | 69 | -1 | 0.04 | 0.49 |
|  | 70 | -1 | 0.05 | -0.20 |
|  | 71 | -1 | 0.55 | -0.20 |
|  | 72 | -1 | -0.55 | 0.49 |
| 19 | 73 | -1.5 | 0.05 | 0.90 |
|  | 74 | -1.5 | 0.05 | 0.30 |
|  | 75 | -1.50 | -0.55 | 0.30 |
|  | 76 | -1.50 | -0.55 | 0.90 |
| 20 | 77 | -2 | 0.05 | 1.48 |
|  | 78 | -2 | 0.05 | 0.88 |
|  | 79 | -2 | -0.55 | 0.88 |
|  | 80 | -2 | -0.55 | 1.48 |
| 21 | 81 | -2.40 | 0.05 | 2.02 |
|  | 82 | -2.40 | 0.05 | 1.42 |
|  | 83 | -2.40 | -0.55 | 1.42 |
|  | 84 | -2.40 | -0.55 | 2.02 |
| 22 | 85 | -2.80 | -0.07 | 2.52 |
|  | 86 | -2.80 | -0.07 | 2.12 |
|  | 87 | -2.80 | -0.42 | 2.12 |
|  | 88 | -2.80 | -0.42 | 2.52 |
| 23 | 89 | -3.56 | -0.19 | 3.40 |
|  | 90 | -3.56 | -0.19 | 3.20 |
|  | 91 | -3.56 | -0.31 | 3.20 |
|  | 92 | -3.46 | -0.31 | 3.40 |
| 24 | 93 | 1.12 | 0.10 | 8.42 |
|  | 94 | 1.12 | -0.60 | 8.42 |
|  | 95 | 1.71 | -0.60 | 7.56 |
|  | 96 | 1.71 | 0.10 | 7.56 |
| 25 | 97 | -0.80 | 0.14 | 2.32 |
|  | 98 | -0.80 | -0.64 | 2.32 |
|  | 99 | -0.81 | -0.64 | 0.54 |
|  | 100 | -0.81 | 0.14 | 0.54 |
| 26 | 101 | -1.04 | 0.22 | 2.71 |
|  | 102 | -1.05 | -0.72 | 2.71 |
|  | 103 | -1.06 | -0.59 | 0.72 |
|  | 104 | -1.06 | 0.09 | 0.72 |
| 27 | 105 | -1.34 | -0.03 | 1 |
|  | 106 | -1.34 | -0.47 | 1 |
|  | 107 | -1.97 | -0.47 | 2.37 |
|  | 108 | -1.97 | -0.03 | 2.37 |
