# Supplementary material for: A Deformable Generic 3D Model of Haptoral Anchor of Monogenean
Source: PLoS One. 2013 Oct 28;8(10):e77650. doi: 10.1371/journal.pone.0077650 (PMC3810373; doi:10.1371/journal.pone.0077650)
Supplement: Table S9 — Cartesian coordinates X, Y & Z for each vertex on the 3D anchor of Dactylogyrus pterocleidus (derived from Transform Properties Window in Blender). (DOC) [file pone.0077650.s009.doc]

**Table S9. Cartesian coordinates X, Y & Z for each vertex on the 3D anchor of *Dactylogyrus pterocleidus* (derived from Transform Properties Window in Blender).**

| Set | Vertices | Coordinates-X | Coordinates-Y | Coordinates-Z |
| --- | --- | --- | --- | --- |
| 1 | 1 | -3.85 | 0.35 | 8.74 |
|  | 2 | -1.82 | 0.35 | 9.55 |
|  | 3 | -1.82 | -0.85 | 9.55 |
|  | 4 | -3.85 | -0.85 | 8.74 |
| 2 | 5 | -3.55 | 0.35 | 8.07 |
|  | 6 | -1.26 | 0.35 | 9.52 |
|  | 7 | -1.26 | -0.85 | 9.52 |
|  | 8 | -3.55 | -0.85 | 8.07 |
| 3 | 9 | -3.11 | 0.59 | 7.18 |
|  | 10 | -0.87 | 0.59 | 9.20 |
|  | 11 | -0.87 | -1.09 | 9.20 |
|  | 12 | -3.11 | -1.09 | 7.18 |
| 4 | 13 | -2.65 | 0.65 | 6.79 |
|  | 14 | -0.62 | 0.59 | 8.87 |
|  | 15 | -0.62 | -1.09 | 8.87 |
|  | 16 | -2.65 | -1.15 | 6.79 |
| 5 | 17 | -2.36 | 0.71 | 6.40 |
|  | 18 | -0.23 | 0.71 | 8.45 |
|  | 19 | -0.23 | -1.21 | 8.45 |
|  | 20 | -2.36 | -1.2 | 6.40 |
| 6 | 21 | -2.06 | 0.68 | 6.24 |
|  | 22 | 0.12 | 0.68 | 8.04 |
|  | 23 | 0.12 | -1.18 | 8.04 |
|  | 24 | -2.06 | -1.18 | 6.24 |
| 7 | 25 | -1.43 | 0.56 | 5.82 |
|  | 26 | 0.40 | 0.56 | 7.55 |
|  | 27 | 0.40 | -1.06 | 7.55 |
|  | 28 | -1.43 | -1.06 | 5.82 |
| 8 | 29 | -0.79 | 0.54 | 5.63 |
|  | 30 | 1.21 | 0.54 | 6.98 |
|  | 31 | 1.21 | -1.04 | 6.98 |
|  | 32 | -0.79 | -1.04 | 5.63 |
| 9 | 33 | -0.46 | 0.59 | 5.25 |
|  | 34 | 3.14 | 0.50 | 6.98 |
|  | 35 | 3.14 | -1 | 6.98 |
|  | 36 | -0.46 | -1.09 | 5.25 |
| 10 | 37 | -0.07 | 0.50 | 5.06 |
|  | 38 | 2.70 | 0.50 | 5.60 |
|  | 39 | 2.70 | -1 | 5.60 |
|  | 40 | -0.07 | -1 | 5.06 |
| 11 | 41 | 0.21 | 0.40 | 4.60 |
|  | 42 | 2.45 | 0.40 | 4.80 |
|  | 43 | 2.45 | -0.90 | 4.80 |
|  | 44 | 0.21 | -0.90 | 4.60 |
| 12 | 45 | 0.34 | 0.40 | 4 |
|  | 46 | 2.08 | 0.40 | 4 |
|  | 47 | 2.08 | -0.90 | 4 |
|  | 48 | 0.34 | -0.90 | 4 |
| 13 | 49 | 0.48 | 0.20 | 3.20 |
|  | 50 | 1.74 | 0.20 | 3.20 |
|  | 51 | 1.74 | -0.70 | 3.20 |
|  | 52 | 0.48 | -0.70 | 3.20 |
| 14 | 53 | 0.32 | 0.20 | 2.50 |
|  | 54 | 1.55 | 0.20 | 2.50 |
|  | 55 | 1.55 | -0.70 | 2.50 |
|  | 56 | 0.32 | -0.70 | 2.50 |
| 15 | 57 | -0.23 | 0.1 | 1.93 |
|  | 58 | 0.71 | 0.10 | 1.04 |
|  | 59 | 0.71 | -0.60 | 1.04 |
|  | 60 | -0.23 | -0.60 | 1.93 |
| 16 | 61 | -0.63 | 0.10 | 1.69 |
|  | 62 | -0.44 | 0.10 | 0.57 |
|  | 63 | -0.44 | -0.60 | 0.57 |
|  | 64 | -0.63 | 0.60 | 1.69 |
| 17 | 65 | -1.25 | 0.10 | 1.48 |
|  | 66 | -1.19 | 0.10 | 0.33 |
|  | 67 | -1.19 | -0.60 | 0.33 |
|  | 68 | -1.25 | -0.60 | 1.48 |
| 18 | 69 | -2.04 | 0 | 1.46 |
|  | 70 | -2.09 | 0 | 0.46 |
|  | 71 | -2.09 | -0.50 | 0.46 |
|  | 72 | -2.04 | -0.50 | 1.46 |
| 19 | 73 | -3.05 | 0 | 1.37 |
|  | 74 | -3.22 | 0 | 0.58 |
|  | 75 | -3.22 | -0.50 | 0.58 |
|  | 76 | -3.05 | -0.50 | 1.37 |
| 20 | 77 | -3.62 | 0 | 1.56 |
|  | 78 | -3.78 | 0 | 0.58 |
|  | 79 | -3.78 | -0.50 | 0.58 |
|  | 80 | -3.62 | -0.50 | 1.56 |
| 21 | 81 | -4.60 | 0 | 1.28 |
|  | 82 | -4.77 | 0 | 0.56 |
|  | 83 | -4.77 | -0.50 | 0.56 |
|  | 84 | -4.60 | -0.50 | 1.28 |
| 22 | 85 | -5.73 | -0.10 | 1.78 |
|  | 86 | -5.90 | -0.10 | 0.85 |
|  | 87 | -5.90 | -0.40 | 0.85 |
|  | 88 | -5.73 | -0.40 | 1.78 |
| 23 | 89 | -6.26 | -0.2 | 2.83 |
|  | 90 | -6.32 | -0.20 | 2.63 |
|  | 91 | -6.32 | -0.30 | 2.63 |
|  | 92 | -6.17 | -0.30 | 2.80 |
| 24 | 93 | 1.42 | 0.22 | 9 |
|  | 94 | 1.42 | -0.72 | 9 |
|  | 95 | 3.58 | -0.72 | 8.63 |
|  | 96 | 3.58 | 0.22 | 8.63 |
| 25 | 97 | -3.71 | 0.02 | 1.72 |
|  | 98 | -3.74 | -0.62 | 1.69 |
|  | 99 | -4.5 | -0.52 | 1.51 |
|  | 100 | -4.49 | 0.12 | 1.54 |
| 26 | 101 | -3.78 | -0.02 | 1.94 |
|  | 102 | -3.79 | -0.57 | 1.93 |
|  | 103 | -4.56 | -0.56 | 1.91 |
|  | 104 | -4.55 | 0.13 | 1.93 |
| 27 | 105 | -4 | -0.17 | 2.16 |
|  | 106 | -4.01 | -0.57 | 2.15 |
|  | 107 | -4.56 | -0.25 | 2.14 |
|  | 108 | -4.55 | 0.15 | 2.14 |
