# Supplementary material for: A Deformable Generic 3D Model of Haptoral Anchor of Monogenean
Source: PLoS One. 2013 Oct 28;8(10):e77650. doi: 10.1371/journal.pone.0077650 (PMC3810373; doi:10.1371/journal.pone.0077650)
Supplement: Table S10 — Cartesian coordinates X, Y & Z for each vertex on the 3D anchor of Dactylogyrus falciunguis (derived from Transform Properties Window in Blender). (DOC) [file pone.0077650.s010.doc]

**Table S10. Cartesian coordinates X, Y & Z for each vertex on the 3D anchor of *Dactylogyrus falciunguis* (derived from Transform Properties Window in Blender).**

| Set | Vertices | Coordinates-X | Coordinates-Y | Coordinates-Z |
| --- | --- | --- | --- | --- |
| 1 | 1 | -2.82 | 0.34 | 10.72 |
|  | 2 | -2.2 | 0.34 | 11.4 |
|  | 3 | -2.21 | -0.84 | 11.4 |
|  | 4 | -2.82 | -0.84 | 10.72 |
| 2 | 5 | -2.09 | 0.34 | 9.81 |
|  | 6 | -1.21 | 0.34 | 10.46 |
|  | 7 | -1.21 | -0.84 | 10.46 |
|  | 8 | -2.09 | -0.84 | 9.81 |
| 3 | 9 | -1.75 | 0.53 | 9.16 |
|  | 10 | -0.57 | 0.58 | 10.25 |
|  | 11 | -0.57 | -1.08 | 10.25 |
|  | 12 | -1.75 | -1.03 | 9.16 |
| 4 | 13 | -1.69 | 0.68 | 8.36 |
|  | 14 | 0.47 | 0.51 | 10.72 |
|  | 15 | 0.47 | -1.01 | 10.72 |
|  | 16 | -1.69 | -1.18 | 8.36 |
| 5 | 17 | -1.16 | 0.93 | 6.74 |
|  | 18 | 1.66 | 1.08 | 11.04 |
|  | 19 | 1.66 | -1.58 | 11.04 |
|  | 20 | -1.32 | -1.43 | 6.77 |
| 6 | 21 | -1.21 | 1.12 | 5.94 |
|  | 22 | 2.22 | 1.18 | 10.60 |
|  | 23 | 2.22 | -1.68 | 10.60 |
|  | 24 | -1.21 | -1.62 | 5.94 |
| 7 | 25 | -1.19 | 1.17 | 5.53 |
|  | 26 | 2.73 | 1.25 | 10.38 |
|  | 27 | 2.73 | -1.75 | 10.38 |
|  | 28 | -1.19 | -1.67 | 5.53 |
| 8 | 29 | -1 | 1.29 | 5.12 |
|  | 30 | 3.15 | 1.33 | 9.744 |
|  | 31 | 3.15 | -1.83 | 9.70 |
|  | 32 | -1 | -1.79 | 5.12 |
| 9 | 33 | -0.9 | 0.84 | 4.84 |
|  | 34 | 2.82 | 1.26 | 5.55 |
|  | 35 | 2.82 | -1.76 | 5.55 |
|  | 36 | -0.90 | -1.34 | 4.84 |
| 10 | 37 | -0.74 | 0.50 | 4.27 |
|  | 38 | 2.21 | 1.12 | 5 |
|  | 39 | 2.21 | -1.62 | 5 |
|  | 40 | -0.74 | -1 | 4.27 |
| 11 | 41 | -0.53 | 0.69 | 3.71 |
|  | 42 | 1.56 | 0.78 | 4.33 |
|  | 43 | 1.56 | -1.28 | 4.33 |
|  | 44 | -0.53 | -1.19 | 3.71 |
| 12 | 45 | -0.53 | 0.63 | 3.27 |
|  | 46 | 1.45 | 0.63 | 3.78 |
|  | 47 | 1.45 | -1.13 | 3.78 |
|  | 48 | -0.53 | -1.13 | 3.27 |
| 13 | 49 | -0.52 | 0.51 | 2.74 |
|  | 50 | 1.29 | 0.59 | 3.21 |
|  | 51 | 1.29 | -1.1 | 3.21 |
|  | 52 | -0.52 | -1.02 | 2.74 |
| 14 | 53 | -0.46 | 0.51 | 2.39 |
|  | 54 | 1.17 | 0.45 | 2.36 |
|  | 55 | 1.17 | -0.94 | 2.36 |
|  | 56 | -0.46 | -1.02 | 2.39 |
| 15 | 57 | -0.33 | 0.23 | 1.70 |
|  | 58 | 1.10 | 0.53 | 2.00 |
|  | 59 | 1.10 | -1.04 | 2.00 |
|  | 60 | -0.33 | -0.74 | 1.70 |
| 16 | 61 | -0.24 | -0.09 | 1.26 |
|  | 62 | 1.13 | 0.66 | 1.70 |
|  | 63 | 1.13 | -1.18 | 1.70 |
|  | 64 | -0.24 | -0.41 | 1.26 |
| 17 | 65 | -0.19 | -0.09 | 0.63 |
|  | 66 | 1.04 | 0.64 | 1.16 |
|  | 67 | 1.04 | -1.14 | 1.16 |
|  | 68 | -0.19 | -0.41 | 0.63 |
| 18 | 69 | -0.17 | 0.18 | 0.41 |
|  | 70 | 0.82 | 0.30 | 0.21 |
|  | 71 | 0.83 | -0.80 | 0.21 |
|  | 72 | -0.17 | -0.68 | 0.41 |
| 19 | 73 | -0.61 | 0.18 | 0.19 |
|  | 74 | -0.31 | 0.18 | -0.82 |
|  | 75 | -0.31 | -0.68 | -0.82 |
|  | 76 | -0.61 | -0.68 | 0.19 |
| 20 | 77 | -1.31 | 0.18 | 0.42 |
|  | 78 | -1.67 | 0.18 | -0.27 |
|  | 79 | -1.67 | -0.68 | -0.27 |
|  | 80 | -1.31 | -0.68 | 0.42 |
| 21 | 81 | -2.61 | 0.14 | 1.30 |
|  | 82 | -2.61 | 0.14 | 0.70 |
|  | 83 | -2.61 | -0.64 | 0.70 |
|  | 84 | -2.61 | -0.64 | 1.30 |
| 22 | 85 | -3.26 | 0.02 | 1.79 |
|  | 86 | -3.42 | 0.02 | 1.32 |
|  | 87 | -3.42 | -0.52 | 1.32 |
|  | 88 | -3.26 | -0.52 | 1.79 |
| 23 | 89 | -5.50 | -0.16 | 3.34 |
|  | 90 | -5.50 | -0.16 | 3.14 |
|  | 91 | -5.50 | -0.34 | 3.14 |
|  | 92 | -5.40 | -0.34 | 3.34 |
| 24 | 93 | -1.90 | -0.01 | 7.22 |
|  | 94 | -1.90 | -0.49 | 7.22 |
|  | 95 | -1.58 | -0.49 | 6.58 |
|  | 96 | -1.58 | -0.01 | 6.58 |
| 25 | 97 | 3.34 | -0.15 | 8.70 |
|  | 98 | 3.34 | -0.35 | 8.70 |
|  | 99 | 3.35 | -0.35 | 7.27 |
|  | 100 | 3.35 | -0.15 | 7.27 |
| 26 | 101 | -0.58 | -0.07 | 1.27 |
|  | 102 | -0.58 | -0.43 | 1.27 |
|  | 103 | -0.84 | -0.50 | 0.33 |
|  | 104 | -0.84 | 0 | 0.33 |
| 27 | 105 | -0.77 | 0 | 1.57 |
|  | 106 | -0.77 | -0.50 | 1.57 |
|  | 107 | -1.62 | -0.50 | 0.83 |
|  | 108 | -1.62 | 0 | 0.83 |
| 28 | 109 | -2.77 | 0.02 | 1.16 |
|  | 110 | -2.77 | -0.52 | 1.16 |
|  | 111 | -1.30 | -0.52 | 2.62 |
|  | 112 | -1.31 | 0.02 | 2.62 |
