# Supplementary material for: A Deformable Generic 3D Model of Haptoral Anchor of Monogenean
Source: PLoS One. 2013 Oct 28;8(10):e77650. doi: 10.1371/journal.pone.0077650 (PMC3810373; doi:10.1371/journal.pone.0077650)
Supplement: Table S11 — Cartesian coordinates X, Y & Z for each vertex on the 3D anchor of Chauhanellus auriculatum (derived from Transform Properties Window in Blender). (DOC) [file pone.0077650.s011.doc]

**Table S11. Cartesian coordinates X, Y & Z for each vertex on the 3D anchor of *Chauhanellus auriculatum* (derived from Transform Properties Window in Blender).**

| Set | Vertices | Coordinates-X | Coordinates-Y | Coordinates-Z |
| --- | --- | --- | --- | --- |
| 1 | 1 | -1.61 | 0.61 | 11.11 |
|  | 2 | -1 | 0.61 | 11.45 |
|  | 3 | -1 | -1.11 | 11.45 |
|  | 4 | -1.61 | -1.11 | 11.11 |
| 2 | 5 | -1.67 | 0.61 | 10.81 |
|  | 6 | -0.59 | 0.61 | 10.85 |
|  | 7 | -0.59 | -1.11 | 10.85 |
|  | 8 | -1.67 | -1.11 | 10.81 |
| 3 | 9 | -1.84 | 0.96 | 9.20 |
|  | 10 | -0.19 | 0.96 | 9.90 |
|  | 11 | -0.19 | -1.46 | 9.90 |
|  | 12 | -1.84 | -1.46 | 9.20 |
| 4 | 13 | -1.71 | 1.20 | 8.26 |
|  | 14 | 0.56 | 0.96 | 9.98 |
|  | 15 | 0.56 | -1.46 | 9.98 |
|  | 16 | -1.71 | -1.70 | 8.26 |
| 5 | 17 | -1.76 | 1.13 | 7.89 |
|  | 18 | -1.26 | 1.33 | 9.36 |
|  | 19 | 1.26 | -1.63 | 9.36 |
|  | 20 | -1.76 | -1.63 | 7.89 |
| 6 | 21 | -1.74 | 1.07 | 7.38 |
|  | 22 | -1.60 | 1.31 | 8.86 |
|  | 23 | 1.60 | -1.81 | 8.86 |
|  | 24 | -1.74 | -1.57 | 7.38 |
| 7 | 25 | -1.63 | 1.09 | 6.82 |
|  | 26 | 2.11 | 1.44 | 8.27 |
|  | 27 | 2.11 | -1.94 | 8.27 |
|  | 28 | -1.63 | -1.59 | 6.82 |
| 8 | 29 | -1.50 | 1.07 | 5.68 |
|  | 30 | 3.03 | 1.42 | 7.58 |
|  | 31 | 3.03 | -1.92 | 7.58 |
|  | 32 | -1.50 | -1.57 | 5.68 |
| 9 | 33 | -1.31 | 1.02 | 4.43 |
|  | 34 | 2.98 | 1.26 | 6.48 |
|  | 35 | 2.98 | -1.76 | 6.48 |
|  | 36 | -1.31 | -1.53 | 4.43 |
| 10 | 37 | -0.93 | 0.92 | 3.91 |
|  | 38 | 2.73 | 0.92 | 4.86 |
|  | 39 | 2.73 | -1.42 | 4.86 |
|  | 40 | -0.93 | -1.42 | 3.91 |
| 11 | 41 | -0.56 | 0.74 | 3.30 |
|  | 42 | 1.91 | 0.74 | 3.59 |
|  | 43 | 1.91 | -1.24 | 3.59 |
|  | 44 | -0.56 | -1.24 | 3.30 |
| 12 | 45 | 0 | 0.62 | 2.60 |
|  | 46 | 1.37 | 0.62 | 2.84 |
|  | 47 | 1.37 | -1.12 | 2.84 |
|  | 48 | 0 | -1.12 | 2.60 |
| 13 | 49 | 0.08 | 0.49 | 1.97 |
|  | 50 | 1.34 | 0.49 | 1.97 |
|  | 51 | 1.34 | -0.99 | 1.97 |
|  | 52 | 0.08 | -0.99 | 1.97 |
| 14 | 53 | 0.45 | 0.49 | 1.04 |
|  | 54 | 1.51 | 0.49 | 1.04 |
|  | 55 | 1.51 | -0.99 | 1.04 |
|  | 56 | 0.45 | -0.99 | 1.04 |
| 15 | 57 | 0.38 | 0.33 | 0.31 |
|  | 58 | 1.42 | 0.33 | 0.31 |
|  | 59 | 1.42 | -0.83 | 0.31 |
|  | 60 | 0.38 | -0.83 | 0.31 |
| 16 | 61 | 0.20 | 0.33 | -0.39 |
|  | 62 | 1.16 | 0.33 | -0.49 |
|  | 63 | 1.16 | -0.83 | -0.49 |
|  | 64 | 0.20 | -0.83 | -0.39 |
| 17 | 65 | -0.17 | 0.33 | -0.96 |
|  | 66 | 0.64 | 0.33 | -1.39 |
|  | 67 | 0.64 | -0.83 | -1.39 |
|  | 68 | -0.17 | -0.83 | -0.96 |
| 18 | 69 | -0.70 | 0.16 | -1.52 |
|  | 70 | -0.29 | 0.16 | -2.11 |
|  | 71 | -0.29 | -0.66 | -2.11 |
|  | 72 | -0.70 | -0.66 | -1.52 |
| 19 | 73 | -1.21 | 0.16 | -1.93 |
|  | 74 | -0.99 | 0.16 | -2.49 |
|  | 75 | -0.99 | -0.66 | -2.49 |
|  | 76 | -1.21 | -0.66 | -1.93 |
| 20 | 77 | -1.67 | 0.16 | -2.30 |
|  | 78 | -1.70 | 0.16 | -2.90 |
|  | 79 | -1.70 | -0.66 | -2.90 |
|  | 80 | -1.67 | -0.66 | -2.30 |
| 21 | 81 | -2.38 | 0.16 | -2.29 |
|  | 82 | -2.41 | 0.16 | -2.89 |
|  | 83 | -2.41 | -0.66 | -2.89 |
|  | 84 | -2.38 | -0.66 | -2.29 |
| 22 | 85 | -3.08 | 0 | -2.23 |
|  | 86 | -3.10 | 0 | -2.63 |
|  | 87 | -3.10 | -0.50 | -2.63 |
|  | 88 | -3.08 | -0.50 | -2.23 |
| 23 | 89 | -4.18 | -0.17 | -1.44 |
|  | 90 | -4.19 | -0.17 | -1.64 |
|  | 91 | -4.19 | -0.33 | -1.64 |
|  | 92 | -4.08 | -0.33 | -1.45 |
| 24 | 93 | -1.94 | -0.08 | 5.26 |
|  | 94 | -1.94 | -0.42 | 5.26 |
|  | 95 | -1.48 | -0.42 | 5.04 |
|  | 96 | -1.48 | -0.08 | 5.04 |
