# Supplementary material for: A Deformable Generic 3D Model of Haptoral Anchor of Monogenean
Source: PLoS One. 2013 Oct 28;8(10):e77650. doi: 10.1371/journal.pone.0077650 (PMC3810373; doi:10.1371/journal.pone.0077650)
Supplement: Table S12 — Cartesian coordinates X, Y & Z for each vertex on the 3D anchor of Chauhanellus caelatus (derived from Transform Properties Window in Blender). (DOC) [file pone.0077650.s012.doc]

**Table S12. Cartesian coordinates X, Y & Z for each vertex on the 3D anchor of *Chauhanellus caelatus* (derived from Transform Properties Window in Blender).**

| Set | Vertices | Coordinate -X | Coordinate- Y | Coordinate-Z |
| --- | --- | --- | --- | --- |
| 1 | 1 | 2.3 | -0.1 | 6.7 |
|  | 2 | 3 | -0.1 | 7.2 |
|  | 3 | 3 | -0.9 | 7.2 |
|  | 4 | 2.3 | -0.9 | 6.7 |
| 2 | 5 | 2.5 | -0.05 | 6.5 |
|  | 6 | 3.4 | -0.05 | 7.1 |
|  | 7 | 3.4 | -0.95 | 7.1 |
|  | 8 | 2.5 | -0.95 | 6.5 |
| 3 | 9 | 3 | 0 | 6.2 |
|  | 10 | 3.8 | 0 | 6.9 |
|  | 11 | 3.8 | -1 | 6.9 |
|  | 12 | 3 | -1 | 6.2 |
| 4 | 13 | 3.4 | 0 | 5.9 |
|  | 14 | 4.2 | 0 | 6.7 |
|  | 15 | 4.2 | -1 | 6.7 |
|  | 16 | 3.4 | -1 | 5.9 |
| 5 | 17 | 3.9 | 0 | 5.6 |
|  | 18 | 4.7 | 0 | 6.5 |
|  | 19 | 4.7 | -1 | 6.5 |
|  | 20 | 3.9 | -1 | 5.6 |
| 6 | 21 | 4.4 | 0 | 5.2 |
|  | 22 | 5.2 | 0 | 6.2 |
|  | 23 | 5.2 | -1 | 6.2 |
|  | 24 | 4.4 | -1 | 5.2 |
| 7 | 25 | 4.6 | 0 | 5 |
|  | 26 | 5.5 | 0 | 6.1 |
|  | 27 | 5.5 | -1 | 6.1 |
|  | 28 | 4.6 | -1 | 5 |
| 8 | 29 | 5 | 0 | 4.8 |
|  | 30 | 5.7 | 0 | 5.8 |
|  | 31 | 5.7 | -1 | 5.8 |
|  | 32 | 5 | -1 | 4.8 |
| 9 | 33 | 5.4 | 0 | 4.5 |
|  | 34 | 7 | 0 | 5.3 |
|  | 35 | 7 | -1 | 5.3 |
|  | 36 | 5.4 | -1 | 4.5 |
| 10 | 37 | 5.6 | 0 | 4.2 |
|  | 38 | 6.6 | 0 | 4.2 |
|  | 39 | 6.6 | -1 | 4.2 |
|  | 40 | 5.6 | -1 | 4.2 |
| 11 | 41 | 5.6 | 0 | 3.8 |
|  | 42 | 6.5 | 0 | 3.8 |
|  | 43 | 6.5 | -1 | 3.8 |
|  | 44 | 5.6 | -1 | 3.8 |
| 12 | 45 | 5.4 | -0.05 | 3.2 |
|  | 46 | 6.3 | -0.05 | 3.2 |
|  | 47 | 6.3 | -0.95 | 3.2 |
|  | 48 | 5.5 | -0.95 | 3.2 |
| 13 | 49 | 5.3 | -0.1 | 2.9 |
|  | 50 | 5.9 | -0.1 | 2.6 |
|  | 51 | 5.9 | -0.9 | 2.6 |
|  | 52 | 5.3 | -0.9 | 2.9 |
| 14 | 53 | 4.8 | -0.1 | 2.5 |
|  | 54 | 5.3 | -0.1 | 2.1 |
|  | 55 | 5.3 | -0.9 | 2.1 |
|  | 56 | 4.8 | -0.9 | 2.5 |
| 15 | 57 | 4.5 | -0.15 | 2.3 |
|  | 58 | 4.8 | -0.15 | 1.8 |
|  | 59 | 4.8 | -0.85 | 1.8 |
|  | 60 | 4.5 | -0.85 | 2.3 |
| 16 | 61 | 4.1 | -0.2 | 2.0 |
|  | 62 | 4.4 | -0.2 | 1.6 |
|  | 63 | 4.4 | -0.8 | 1.6 |
|  | 64 | 4.1 | -0.8 | 2.0 |
| 17 | 65 | 3.6 | -0.2 | 2.0 |
|  | 66 | 3.8 | -0.2 | 1.5 |
|  | 67 | 3.8 | -0.8 | 1.5 |
|  | 68 | 3.6 | -0.8 | 2.0 |
| 18 | 69 | 3 | -0.25 | 1.6 |
|  | 70 | 3.1 | -0.25 | 1.1 |
|  | 71 | 3.1 | -0.75 | 1.1 |
|  | 72 | 3 | -0.75 | 1.6 |
| 19 | 73 | 2.6 | -0.25 | 1.4 |
|  | 74 | 2.6 | -0.25 | 1.0 |
|  | 75 | 2.8 | -0.75 | 1.0 |
|  | 76 | 2.8 | -0.75 | 1.4 |
| 20 | 77 | 2.1 | -0.25 | 1.2 |
|  | 78 | 2.1 | -0.25 | 0.8 |
|  | 79 | 2.1 | -0.75 | 0.8 |
|  | 80 | 2.1 | -0.75 | 1.2 |
| 21 | 81 | 1.6 | -0.3 | 1.1 |
|  | 82 | 1.6 | -0.3 | 0.8 |
|  | 83 | 1.6 | -0.7 | 0.8 |
|  | 84 | 1.6 | -0.7 | 1.1 |
| 22 | 85 | 1.1 | -0.4 | 1.1 |
|  | 86 | 1.1 | -0.4 | 0.9 |
|  | 87 | 1.1 | -0.6 | 0.9 |
|  | 88 | 1.1 | -0.6 | 1.1 |
| 23 | 89 | 0.8 | -0.4 | 1.5 |
|  | 90 | 0.7 | -0.4 | 1.4 |
|  | 91 | 0.7 | -0.6 | 1.4 |
|  | 92 | 0.8 | -0.6 | 1.5 |
| 24 | 93 | 6.8 | 0 | 7.4 |
|  | 94 | 7.5 | 0 | 7.2 |
|  | 95 | 7.5 | -1 | 7.2 |
|  | 96 | 6.8 | -1 | 7.4 |
