# Supplementary material for: A Deformable Generic 3D Model of Haptoral Anchor of Monogenean
Source: PLoS One. 2013 Oct 28;8(10):e77650. doi: 10.1371/journal.pone.0077650 (PMC3810373; doi:10.1371/journal.pone.0077650)
Supplement: Table S13 — Cartesian coordinates X, Y & Z for each vertex on the 3D anchor of Squalonchocotyle mitsukurii (derived from Transform Properties Window in Blender). (DOC) [file pone.0077650.s013.doc]

**Table S13. Cartesian coordinates X, Y & Z for each vertex on the 3D anchor of *Squalonchocotyle mitsukurii* (derived from Transform Properties Window in Blender).**

| Set | Vertices | Coordinate -X | Coordinate- Y | Coordinate-Z |
| --- | --- | --- | --- | --- |
| 1 | 1 | -3.31 | -3.10 | -0.77 |
|  | 2 | -3.30 | -3.10 | -0.94 |
|  | 3 | -3.53 | -3.20 | -0.85 |
|  | 4 | -3.53 | -3.20 | -0.69 |
| 2 | 5 | -3.21 | -1.07 | -2.37 |
|  | 6 | -3.49 | -0.90 | -3.83 |
|  | 7 | -4.32 | -0.61 | -3.73 |
|  | 8 | -4.11 | -0.79 | -2.25 |
| 3 | 9 | -2.98 | 0.46 | -2.80 |
|  | 10 | -2.93 | 1.52 | -3.23 |
|  | 11 | -4.27 | 1.92 | -3.06 |
|  | 12 | -4.24 | 0.85 | -2.37 |
| 4 | 13 | -2.12 | 1.20 | -0.69 |
|  | 14 | -2.57 | 2.99 | -2.34 |
|  | 15 | -3.82 | 3.27 | -2.02 |
|  | 16 | -3.75 | 1.49 | -0.35 |
| 5 | 17 | -2.05 | 1.91 | -0.16 |
|  | 18 | -2.21 | 4.26 | -1.28 |
|  | 19 | -3.62 | 4.32 | -0.78 |
|  | 20 | -3.60 | 2.24 | 0.24 |
| 6 | 21 | -1.61 | 2.94 | 0.74 |
|  | 22 | -1.57 | 5.97 | -0.07 |
|  | 23 | -3.27 | 6.04 | 0.35 |
|  | 24 | -3.33 | 3.31 | 1.15 |
| 7 | 25 | -1.01 | 4.30 | 1.98 |
|  | 26 | -0.85 | 7.42 | 2.30 |
|  | 27 | -2.64 | 7.46 | 2.94 |
|  | 28 | -2.79 | 4.34 | 2.62 |
| 8 | 29 | -0.34 | 4.89 | 3.32 |
|  | 30 | -0.08 | 7.90 | 3.88 |
|  | 31 | -2.40 | 7.98 | 4.82 |
|  | 32 | -2.66 | 5.35 | 4.33 |
| 9 | 33 | 0.36 | 5.71 | 5.01 |
|  | 34 | 0.69 | 8.20 | 5.78 |
|  | 35 | -2.07 | 8.24 | 6.75 |
|  | 36 | -2.39 | 5.77 | 5.95 |
| 10 | 37 | 0.64 | 5.73 | 6.19 |
|  | 38 | 0.95 | 7.95 | 6.86 |
|  | 39 | -1.81 | 8.10 | 7.49 |
|  | 40 | -2.13 | 5.86 | 6.82 |
| 11 | 41 | 0.94 | 5.68 | 7.62 |
|  | 42 | 1.15 | 7.85 | 8.07 |
|  | 43 | -1.44 | 7.89 | 8.46 |
|  | 44 | -1.66 | 5.72 | 7.96 |
| 12 | 45 | 1.34 | 5.56 | 8.62 |
|  | 46 | 1.59 | 7.25 | 9.28 |
|  | 47 | -0.74 | 7.30 | 10.11 |
|  | 48 | -0.98 | 5.29 | 9.45 |
| 13 | 49 | 1.80 | 4.66 | 10.29 |
|  | 50 | 1.51 | 6.39 | 10.87 |
|  | 51 | -0.17 | 6.43 | 11.46 |
|  | 52 | -0.36 | 4.70 | 10.90 |
| 14 | 53 | 1.93 | 3.47 | 11.45 |
|  | 54 | 1.96 | 5.27 | 11.93 |
|  | 55 | 0.36 | 5.31 | 12.50 |
|  | 56 | 0.16 | 3.51 | 12.02 |
| 15 | 57 | 1.89 | 2.32 | 12.25 |
|  | 58 | 2.13 | 4.19 | 12.83 |
|  | 59 | 0.76 | 4.23 | 13.31 |
|  | 60 | 0.51 | 2.36 | 12.73 |
| 16 | 61 | 1.92 | 1.64 | 12.49 |
|  | 62 | 2.25 | 2.79 | 13.60 |
|  | 63 | 1.00 | 2.83 | 14.04 |
|  | 64 | 0.67 | 1.68 | 12.94 |
| 17 | 65 | 1.95 | 0.29 | 12.93 |
|  | 66 | 2.33 | 0.87 | 14.10 |
|  | 67 | 1.08 | 0.91 | 14.55 |
|  | 68 | 0.70 | 0.34 | 13.37 |
| 18 | 69 | 1.98 | -0.35 | 12.91 |
|  | 70 | 2.31 | -0.72 | 14.22 |
|  | 71 | 1.06 | -0.85 | 14.64 |
|  | 72 | 0.73 | -0.48 | 13.33 |
| 19 | 73 | 2.13 | -1.18 | 12.96 |
|  | 74 | 2.43 | -1.71 | 14.02 |
|  | 75 | 1.18 | -1.84 | 14.44 |
|  | 76 | 0.88 | -1.31 | 13.38 |
| 20 | 77 | 1.96 | -2.23 | 12.61 |
|  | 78 | 2.27 | -2.67 | 13.54 |
|  | 79 | 1.11 | -2.69 | 13.93 |
|  | 80 | 0.79 | -2.24 | 13.00 |
| 21 | 81 | 1.75 | -3.08 | 11.98 |
|  | 82 | 1.80 | -3.53 | 12.97 |
|  | 83 | 0.75 | -3.33 | 13.34 |
|  | 84 | 0.44 | -2.89 | 12.34 |
| 22 | 85 | 0.96 | -4.07 | 11.25 |
|  | 86 | 1.53 | -4.28 | 12.03 |
|  | 87 | 0.58 | -4.16 | 12.28 |
|  | 88 | 0.18 | -3.78 | 11.57 |
| 23 | 89 | 0.79 | -4.75 | 10.25 |
|  | 90 | 1.05 | -5.11 | 10.88 |
|  | 91 | 0.30 | -5.02 | 11.22 |
|  | 92 | 0.17 | -4.25 | 10.43 |
| 24 | 93 | 0.80 | 6.40 | 11.12 |
|  | 94 | 1.22 | 5.11 | 12.12 |
